# Supplementary material for: Copy number footprints of platinum-based anticancer therapies
Source: PLoS Genet. 2023 Feb 13;19(2):e1010634. doi: 10.1371/journal.pgen.1010634 (PMC9956877; doi:10.1371/journal.pgen.1010634)
Supplement: S1 Fig — Primary and metastatic WGD tumors a) Separation between WGD and non WGD tumors across the primary (PCAWG) and metastatic (HMF) cohorts on the basis of their ploidy and level of homozygosity. Similar to Fig 1A, but with the tumors of the two cohorts in separate panels. b-i) Distribution of ploidy (left) and fraction of the genome with LOH (right) of WGD tumors of different cancer types across primary and metastatic cohorts. Similar to Fig 1C, but separated by cancer types represented in both, PCAWG and HMF cohorts. The boxes inside the violin plots delimit the first, second and third quartiles of the distribution. All tumors in each group are represented as dots. P-values were derived from a two-tailed Wilcoxon-Mann-Whitney test. (PDF) [file pgen.1010634.s001.pdf]

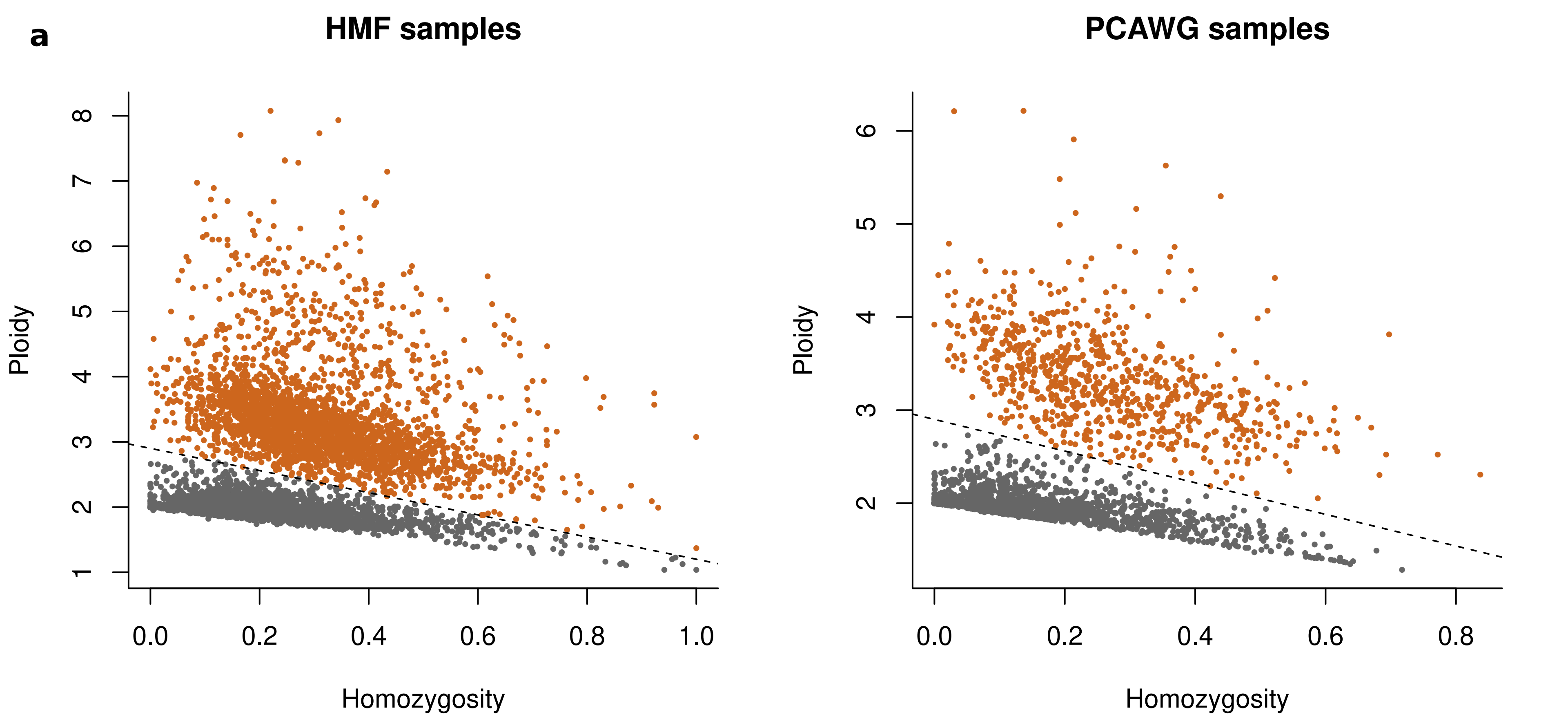
a
HMF samples
PCAWG samples
Ploidy
Homozygosity
1
2
3
4
5
6
7
8
0.0
0.2
0.4
0.6
0.8
1.0
Ploidy
Homozygosity
2
3
4
5
6
0.0
0.2
0.4
0.6
0.8

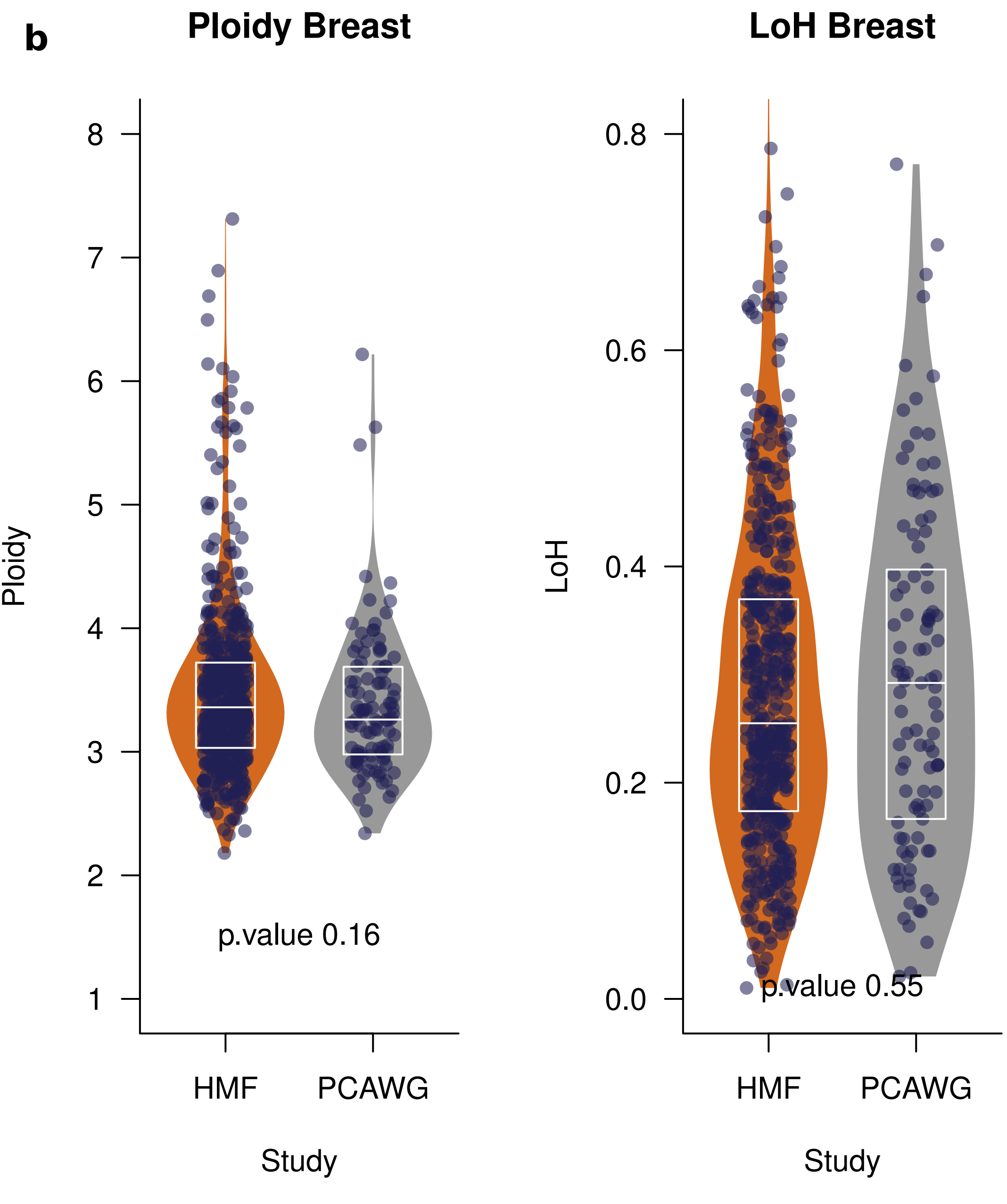
b
Ploidy Breast
LoH Breast
Ploidy
8
7
6
5
4
3
2
1
p.value 0.16
HMF
PCAWG
Study
LoH
0.8
0.6
0.4
0.2
0.0
p.value 0.55
HMF
PCAWG
Study

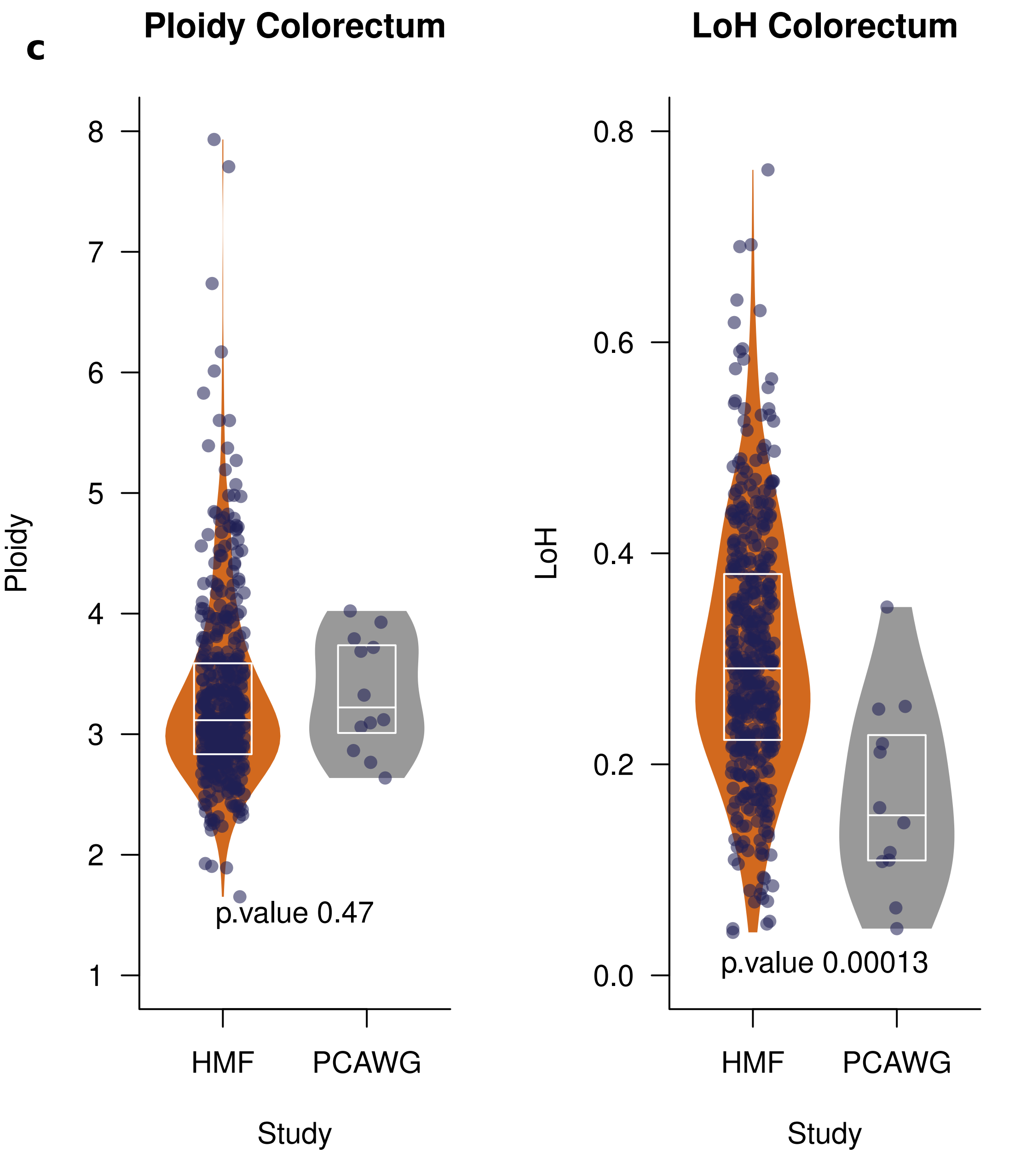
c
Ploidy Colorectum
Ploidy
8
7
6
5
4
3
2
1
p.value 0.47
HMF
PCAWG
Study
LoH Colorectum
LoH
0.8
0.6
0.4
0.2
0.0
p.value 0.00013
HMF
PCAWG
Study

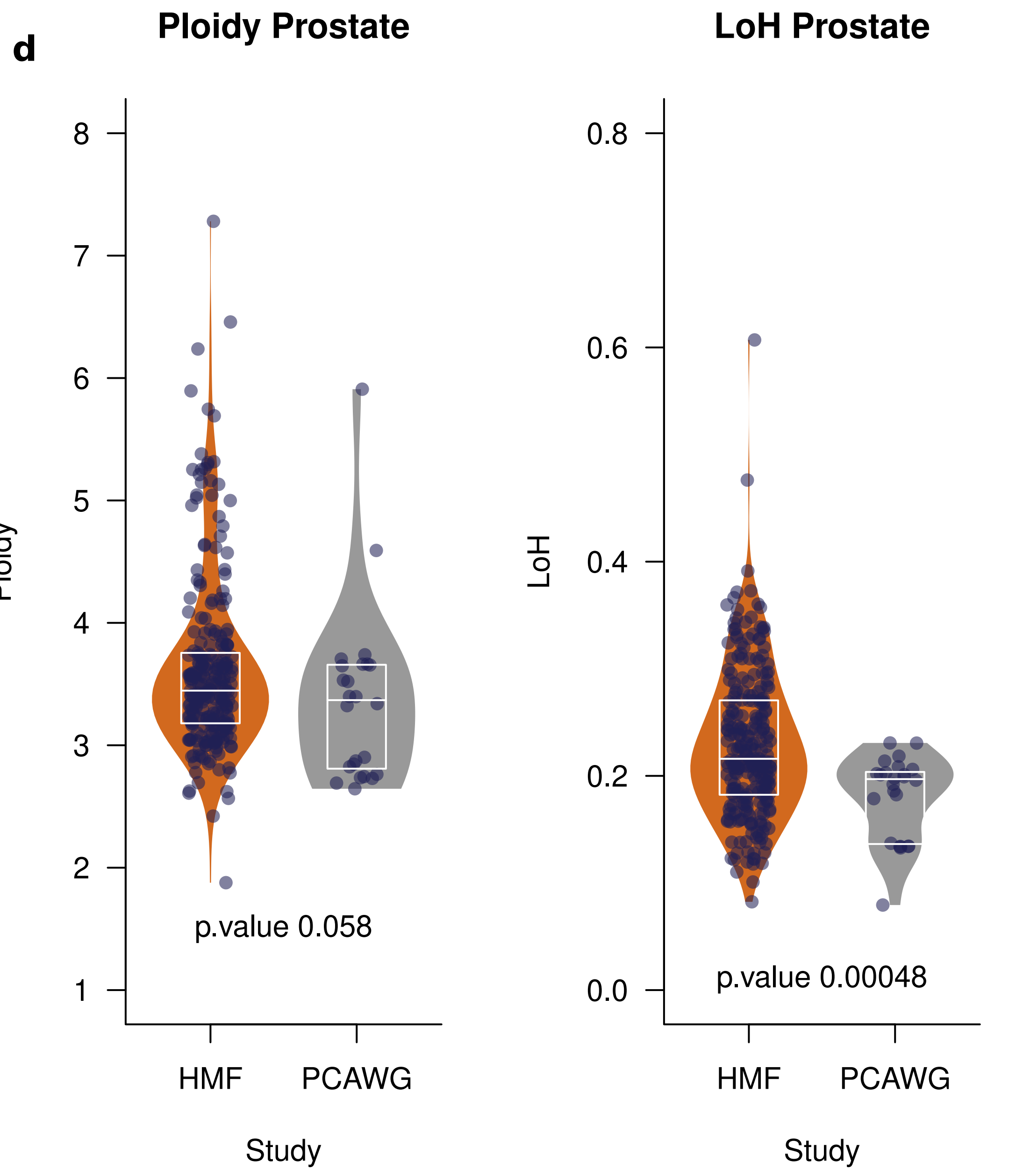
d
Ploidy Prostate
LoH Prostate
Ploidy
8
7
6
5
4
3
2
1
p.value 0.058
HMF
PCAWG
Study
LoH
0.8
0.6
0.4
0.2
0.0
p.value 0.00048
HMF
PCAWG
Study

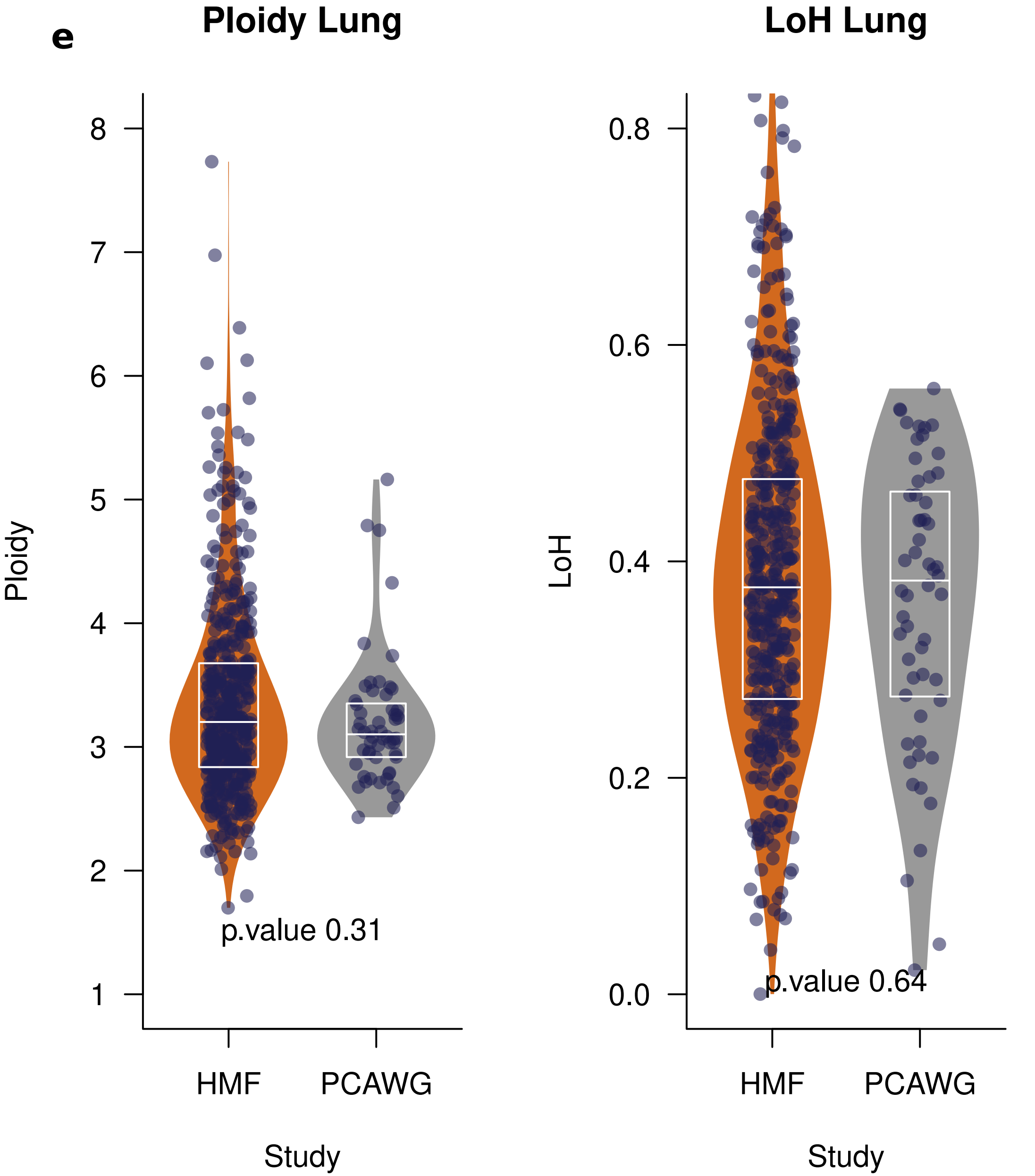

e
Ploidy Lung
LoH Lung
Ploidy
8
7
6
5
4
3
2
1
p.value 0.31
HMF
PCAWG
Study
LoH
0.8
0.6
0.4
0.2
0.0
p.value 0.64
HMF
PCAWG
Study

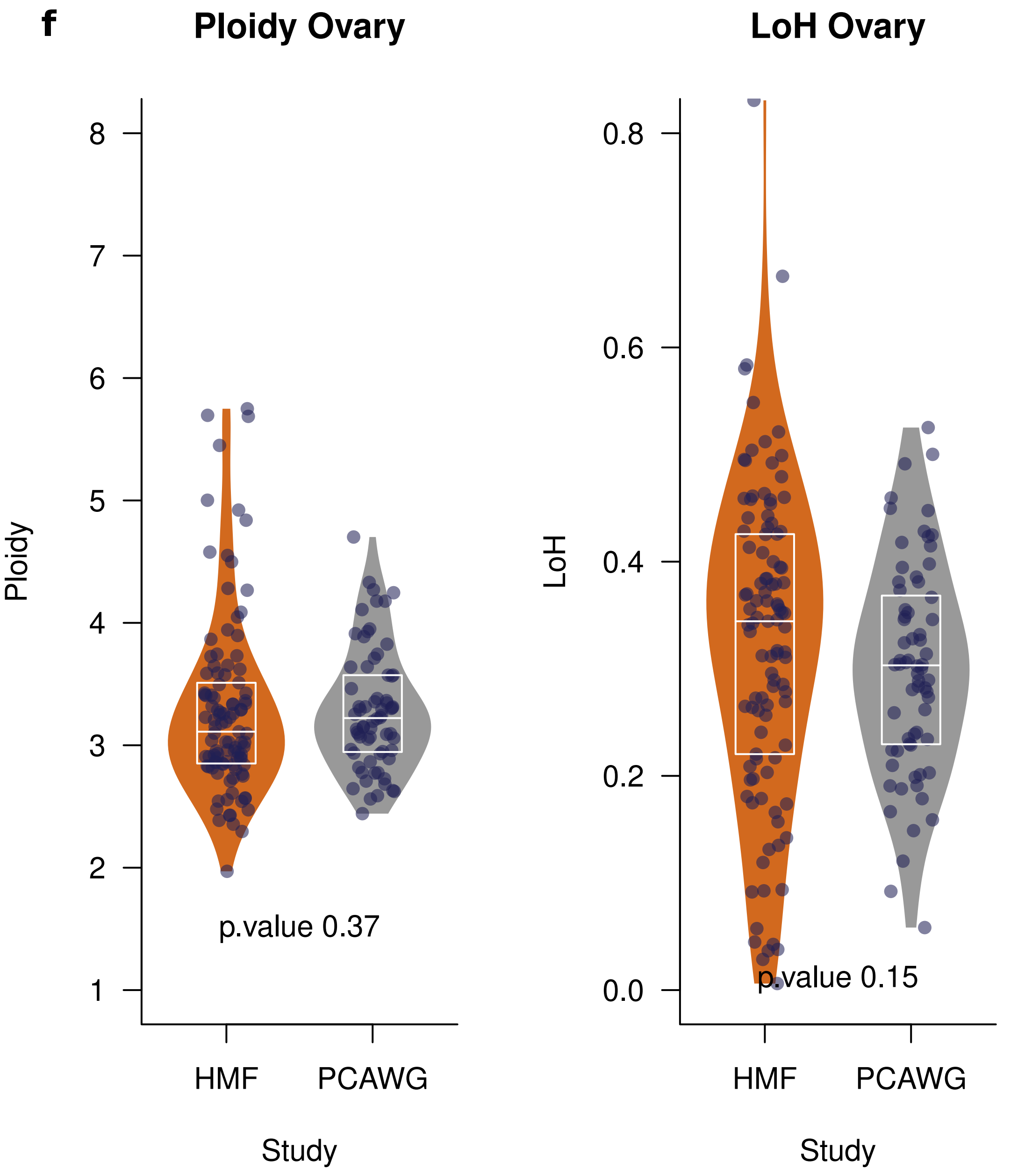
f
Ploidy Ovary
Ploidy
8
7
6
5
4
3
2
1
p.value 0.37
HMF
PCAWG
Study
LoH Ovary
LoH
0.8
0.6
0.4
0.2
0.0
p.value 0.15
HMF
PCAWG
Study

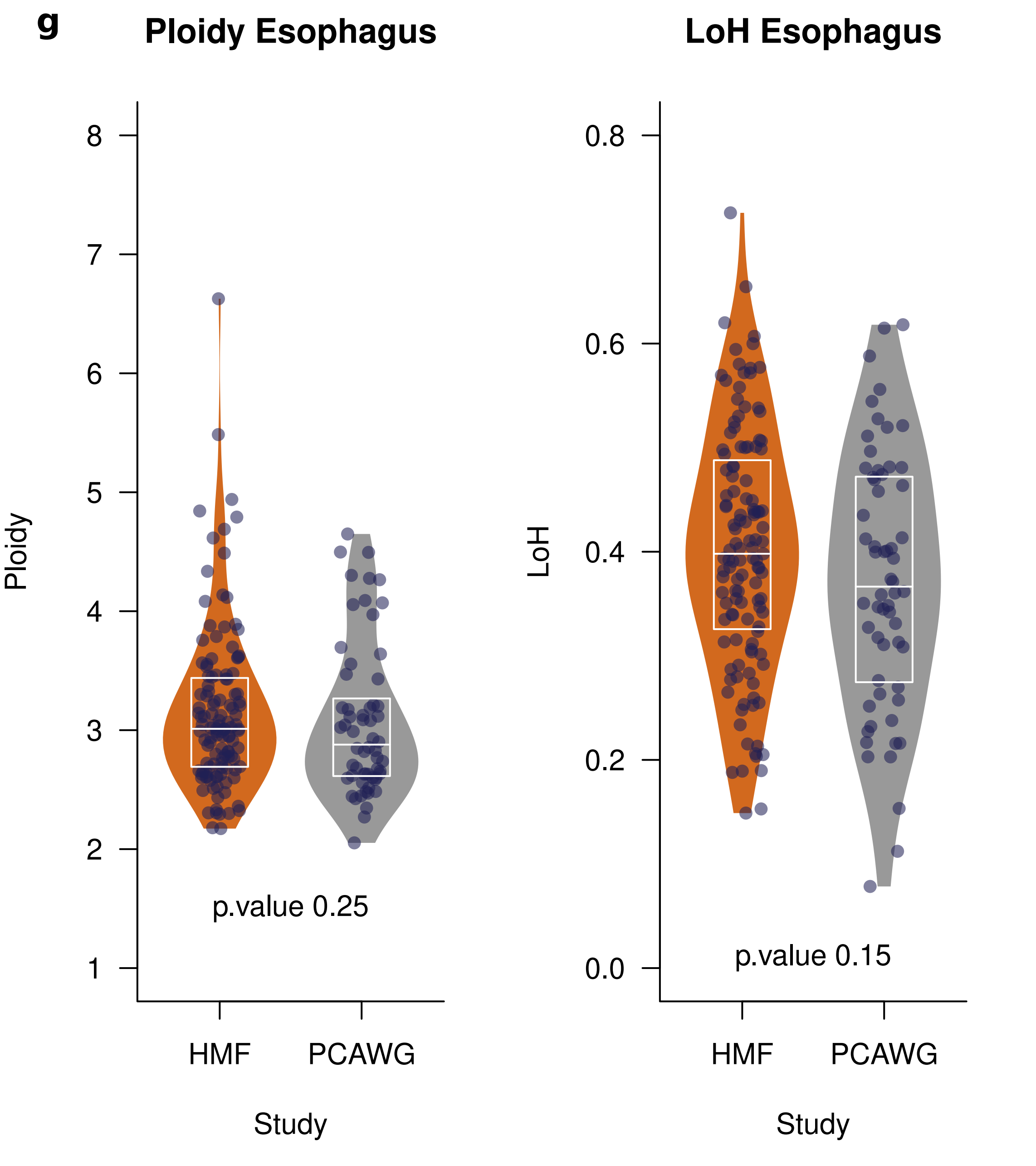
g
Ploidy Esophagus
LoH Esophagus
Ploidy
8
7
6
5
4
3
2
1
p.value 0.25
HMF
PCAWG
Study
LoH
0.8
0.6
0.4
0.2
0.0
p.value 0.15
HMF
PCAWG
Study

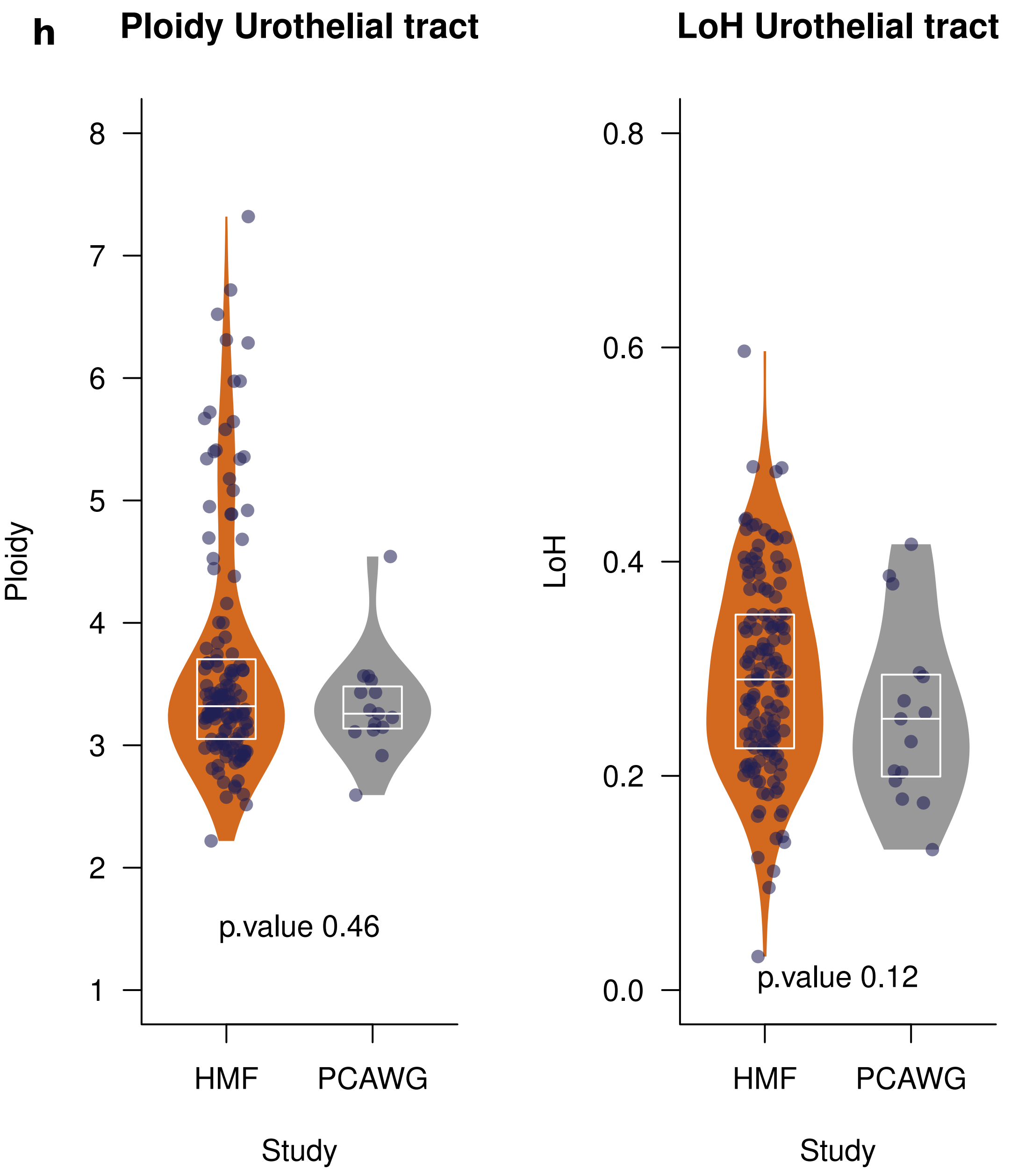
h
Ploidy Urothelial tract
Ploidy
8
7
6
5
4
3
2
1
p.value 0.46
HMF
PCAWG
Study
LoH Urothelial tract
LoH
0.8
0.6
0.4
0.2
0.0
p.value 0.12
HMF
PCAWG
Study

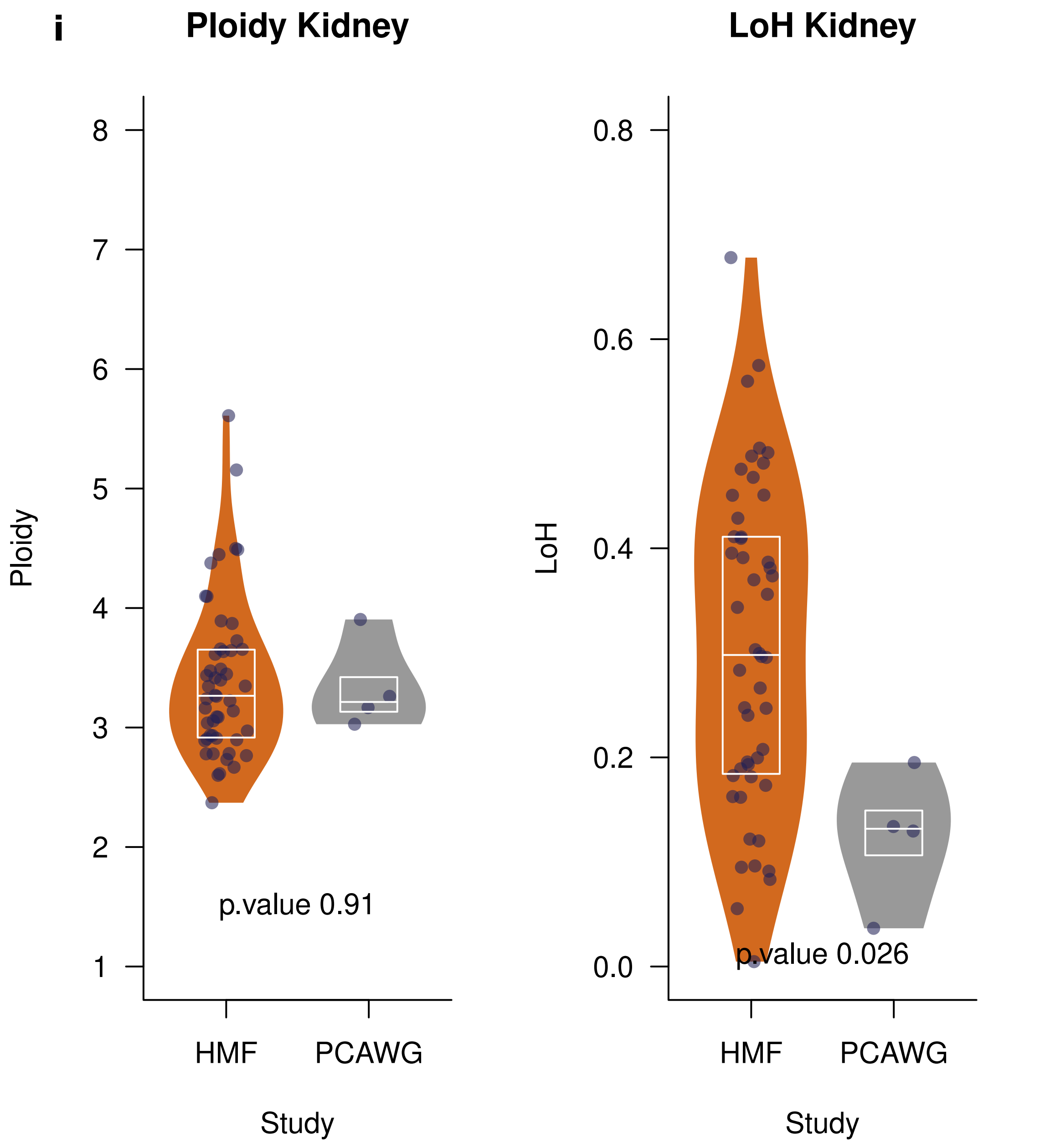
i
Ploidy Kidney
Ploidy
8
7
6
5
4
3
2
1
p.value 0.91
HMF
PCAWG
Study
LoH Kidney
LoH
0.8
0.6
0.4
0.2
0.0
p.value 0.026
HMF
PCAWG
Study
