# Supplementary material for: Copy number footprints of platinum-based anticancer therapies
Source: PLoS Genet. 2023 Feb 13;19(2):e1010634. doi: 10.1371/journal.pgen.1010634 (PMC9956877; doi:10.1371/journal.pgen.1010634)
Supplement: S2 Fig — Significant comparisons (uncorrected p-value below 0.05) are highlighted with asterisks below each bar. (PDF) [file pgen.1010634.s002.pdf]

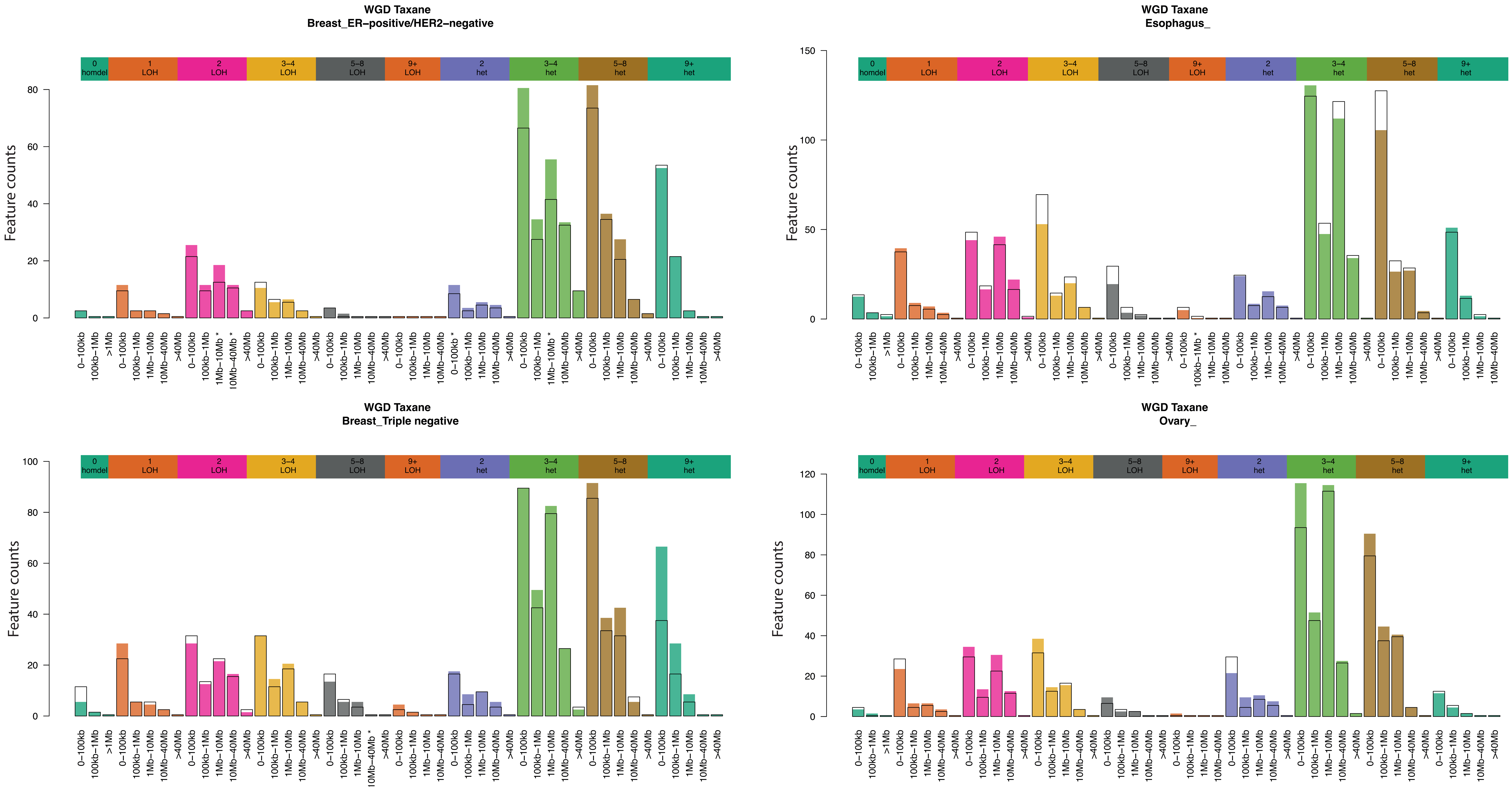

WGD Taxane
Breast_ER−positive/HER2−negative
WGD Taxane
Esophagus_
WGD Taxane
Breast_Triple negative
WGD Taxane
Ovary_
Feature counts
0 homdel
1 LOH
2 LOH
3−4 LOH
5−8 LOH
9+ LOH
2 het
3−4 het
5−8 het
9+ het
0−100kb
100kb−1Mb
>1Mb
1Mb−10Mb
10Mb−40Mb
>40Mb
0−100kb *
1Mb−10Mb *
10Mb−40Mb *
100kb−1Mb *
