# Supplementary material for: Copy number footprints of platinum-based anticancer therapies
Source: PLoS Genet. 2023 Feb 13;19(2):e1010634. doi: 10.1371/journal.pgen.1010634 (PMC9956877; doi:10.1371/journal.pgen.1010634)
Supplement: S3 Fig — Comparison with CN signatures a) Comparison of the activity of CN signatures extracted de novo from HMF WGD tumors between samples exposed and unexposed to different anticancer treatments. No signature appears with significantly different activity between exposed and unexposed WGD tumors. b) Equivalence (linear combination reconstruction) between CN signatures extracted de novo from the HMF cohort and CN signatures previously extracted from primary tumors (ref. 21). c) Comparison of the activity of CN signatures extracted from primary tumors (ref. 21) between samples exposed and unexposed to different anticancer treatments. No signature appears with significantly different activity between exposed and unexposed WGD tumors in the HMF cohort. (PDF) [file pgen.1010634.s003.pdf]

**a**

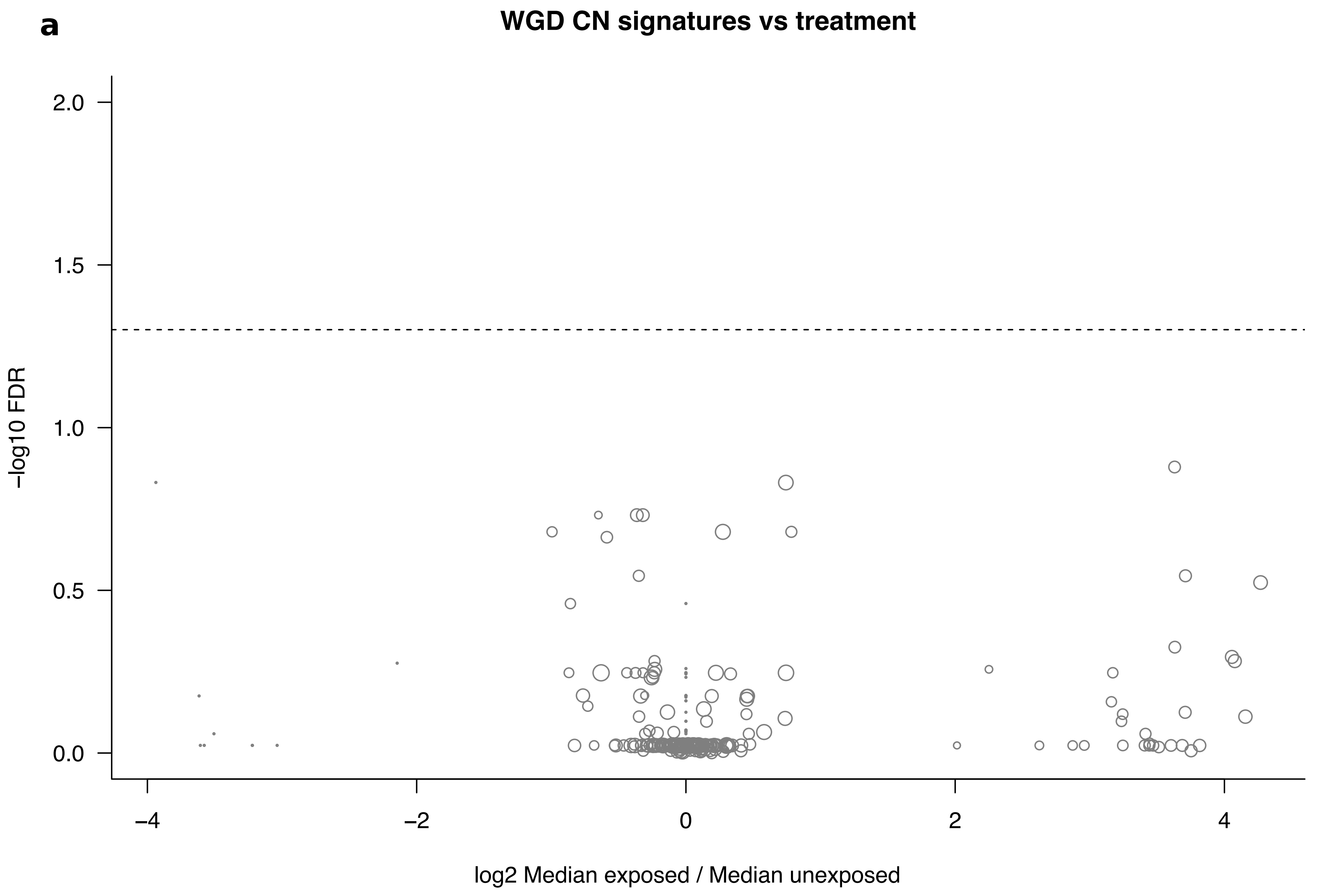
WGD CN signatures vs treatment
2.0
1.5
1.0
0.5
0.0
−log10 FDR
−4
−2
0
2
4
log2 Median exposed / Median unexposed

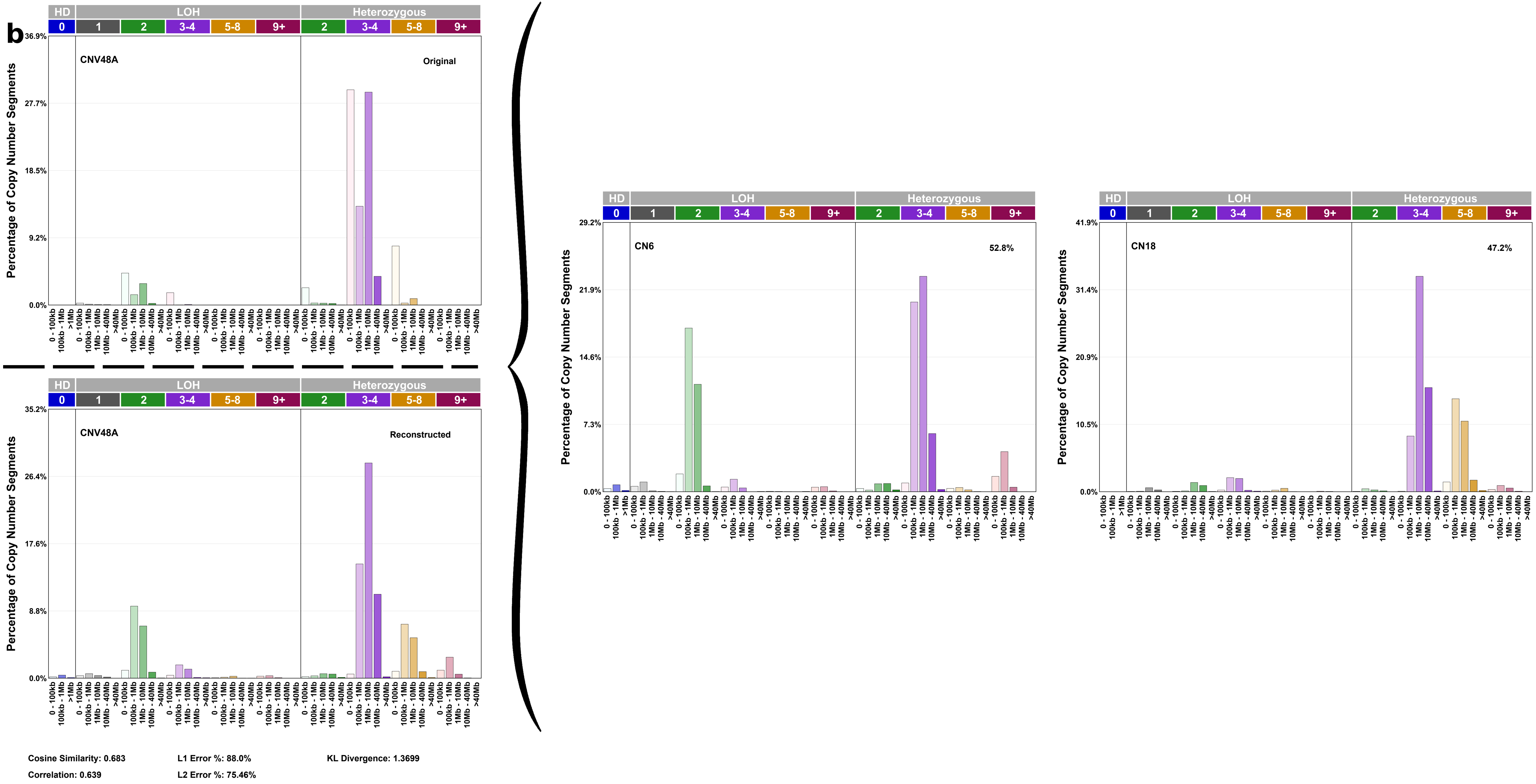
b
HD
LOH
Heterozygous
0
1
2
3-4
5-8
9+
CNV48A
Original
Reconstructed
Percentage of Copy Number Segments
36.9%
27.7%
18.5%
9.2%
0.0%
35.2%
26.4%
17.6%
8.8%
0.0%
0 - 100kb
100kb - 1Mb
>1Mb
1Mb - 10Mb
10Mb - 40Mb
>40Mb
CN6
52.8%
29.2%
21.9%
14.6%
7.3%
CN18
47.2%
41.9%
31.4%
20.9%
10.5%
Cosine Similarity: 0.683
Correlation: 0.639
L1 Error %: 88.0%
L2 Error %: 75.46%
KL Divergence: 1.3699

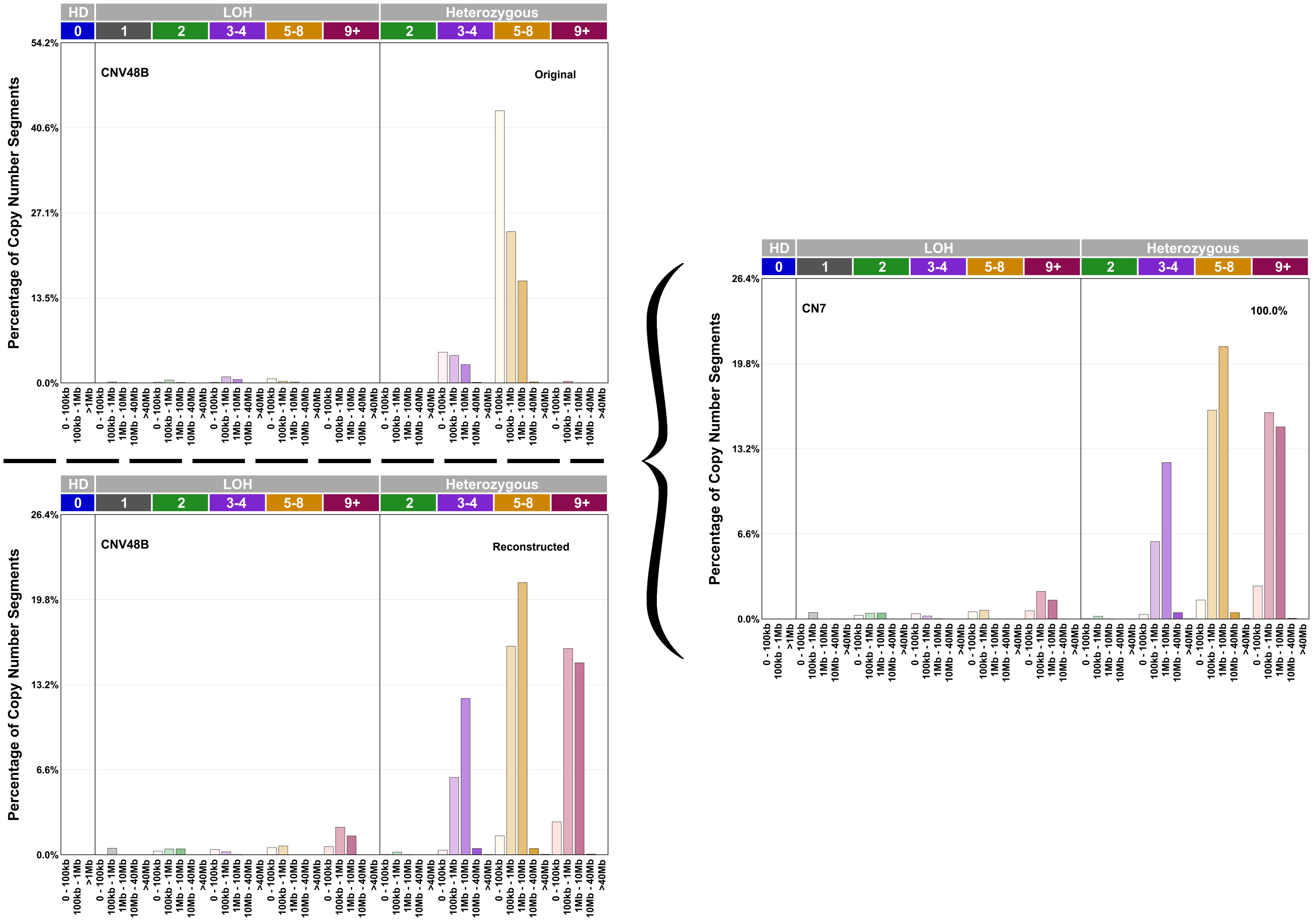


Cosine Similarity: 0.441

Correlation: 0.378

L1 Error %: 112.0%

L2 Error %: 92.985%

KL Divergence: 1000.0

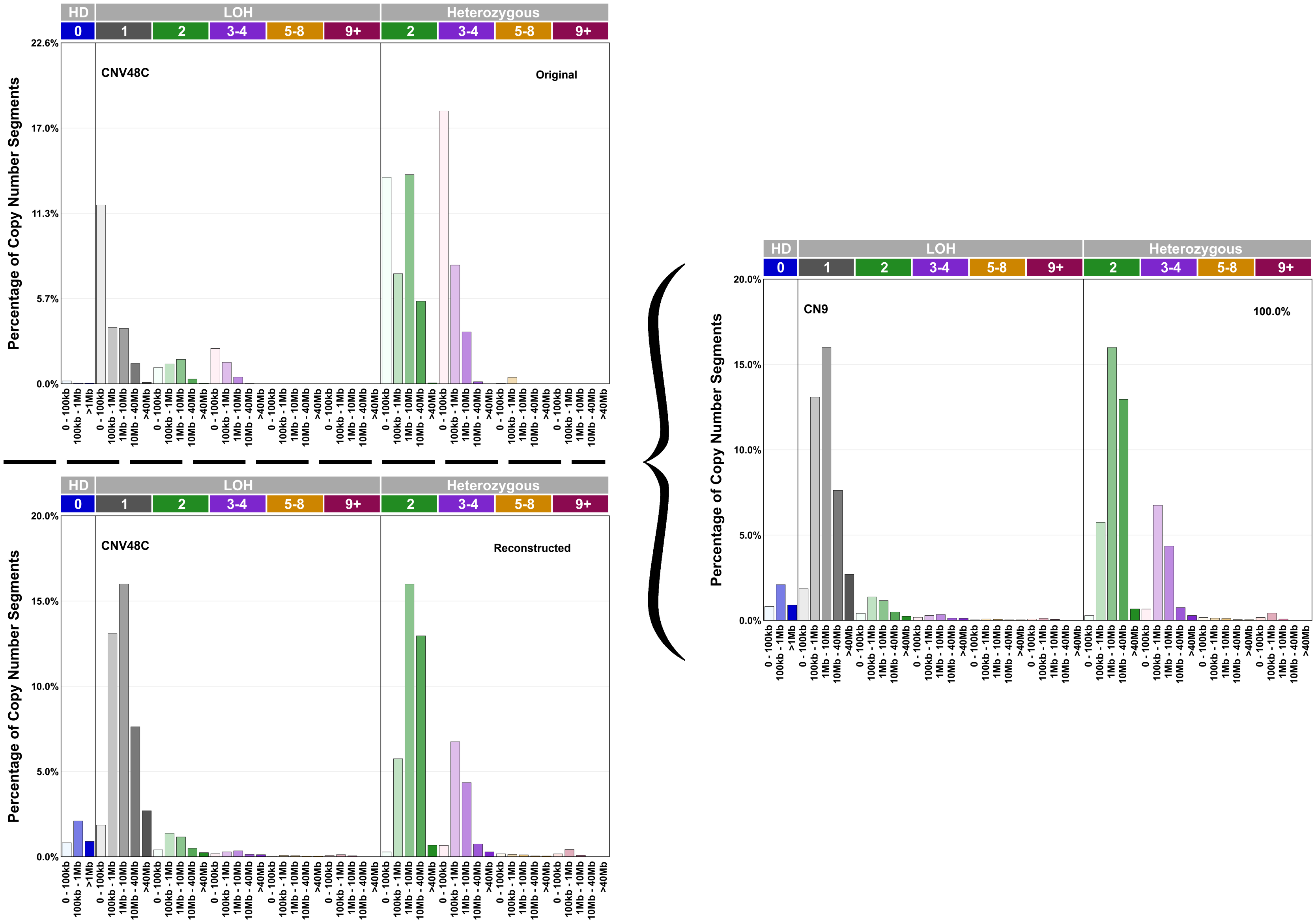


Cosine Similarity: 0.544　　L1 Error %: 97.0%　　KL Divergence: 1.2671

Correlation: 0.429　　L2 Error %: 95.759%

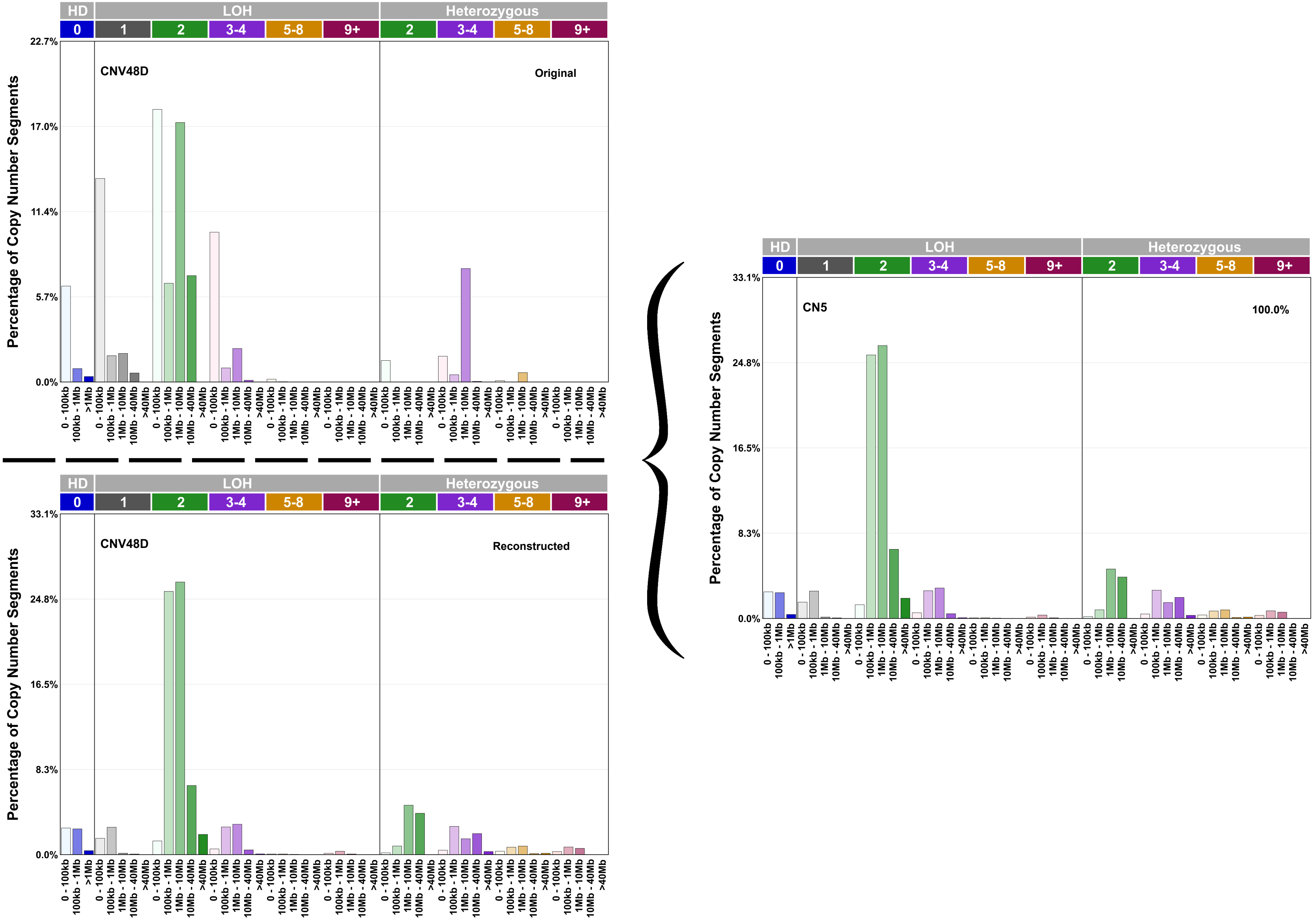


Cosine Similarity: 0.595
L1 Error %: 107.0%
KL Divergence: 1.1467

Correlation: 0.518
L2 Error %: 98.471%

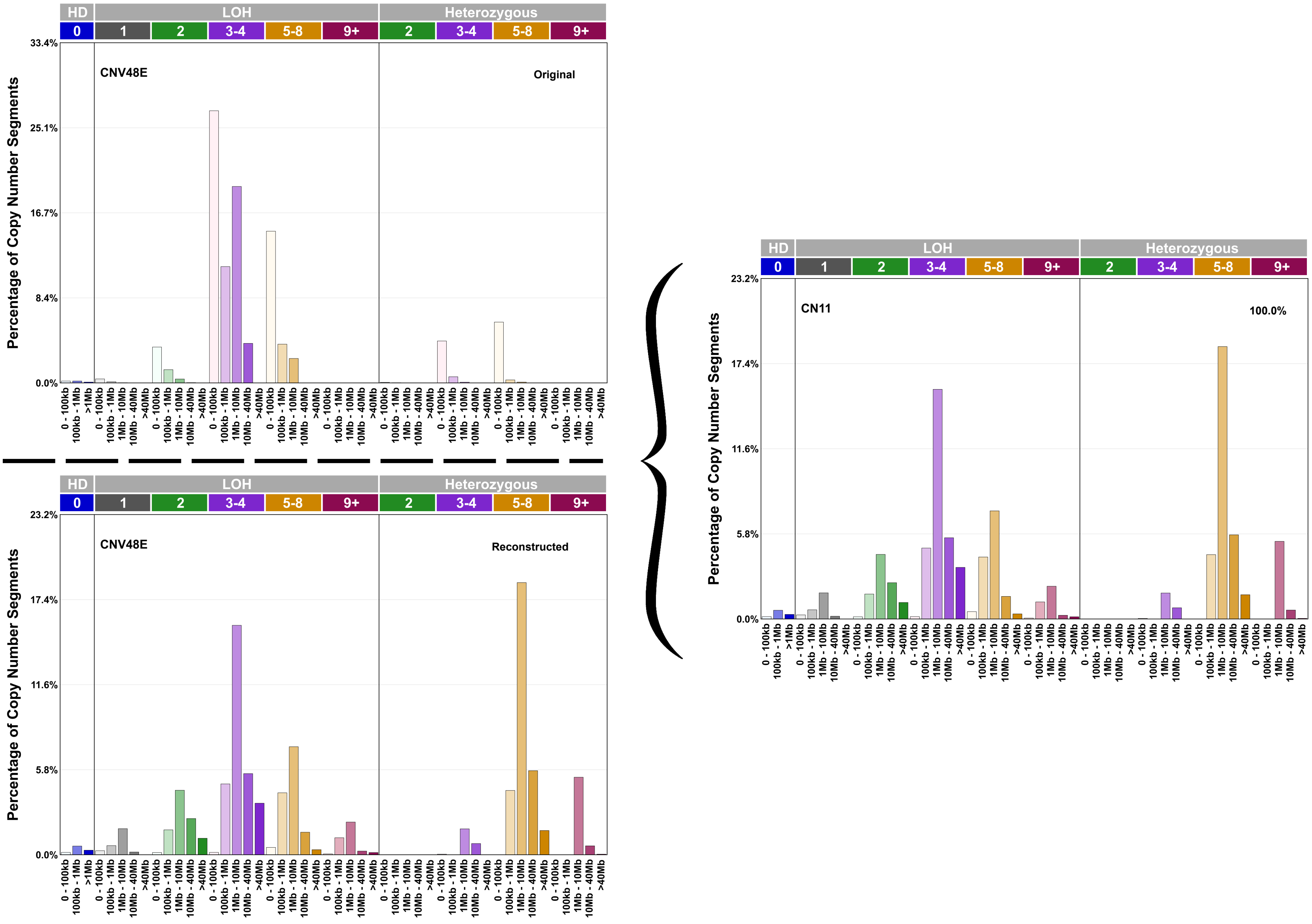


Cosine Similarity: 0.376 L1 Error %: 131.0% KL Divergence: 1000.0

Correlation: 0.24 L2 Error %: 99.226%

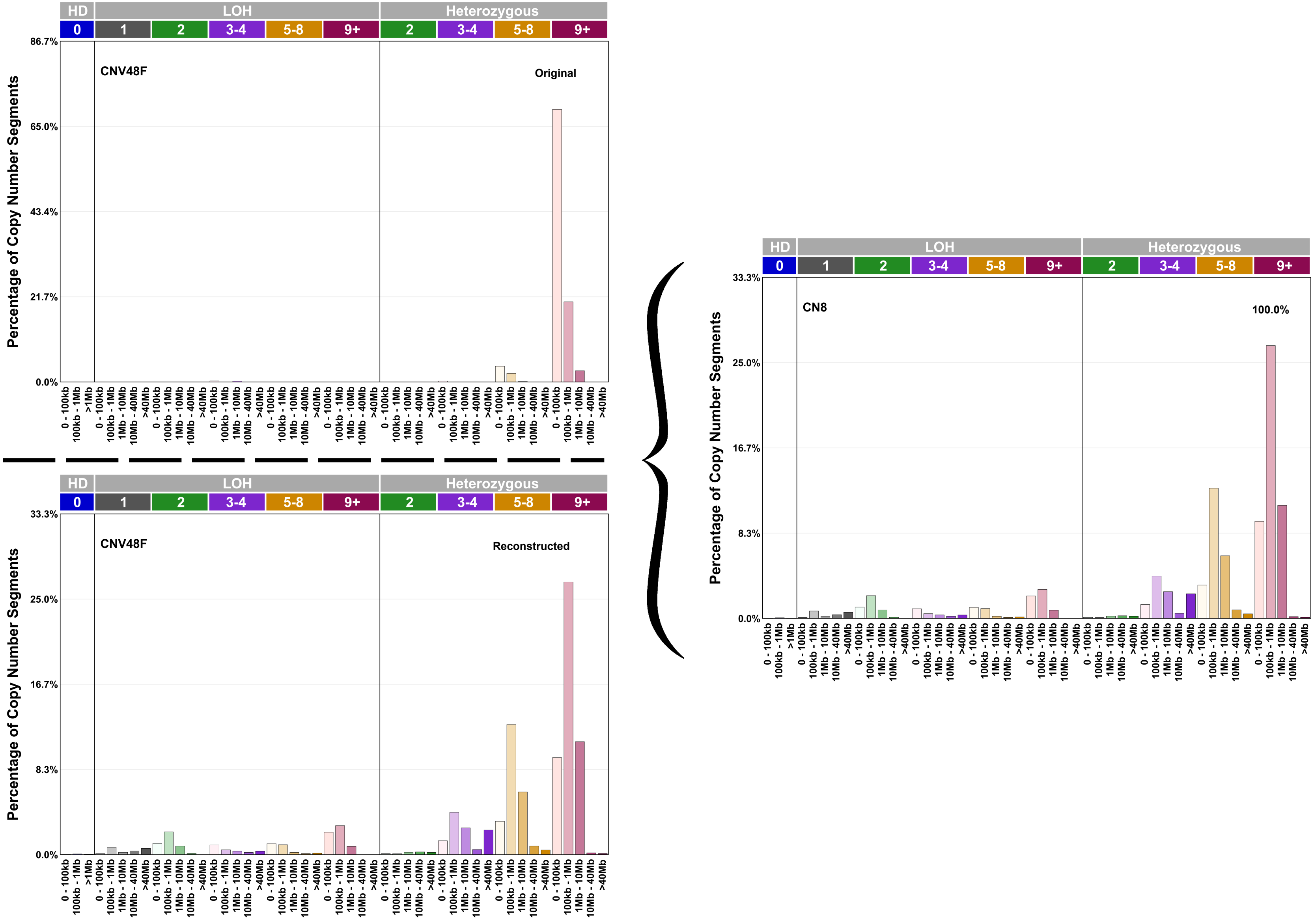


Cosine Similarity: 0.51 L1 Error %: 121.0% KL Divergence: 1.2369

Correlation: 0.48 L2 Error %: 85.486%

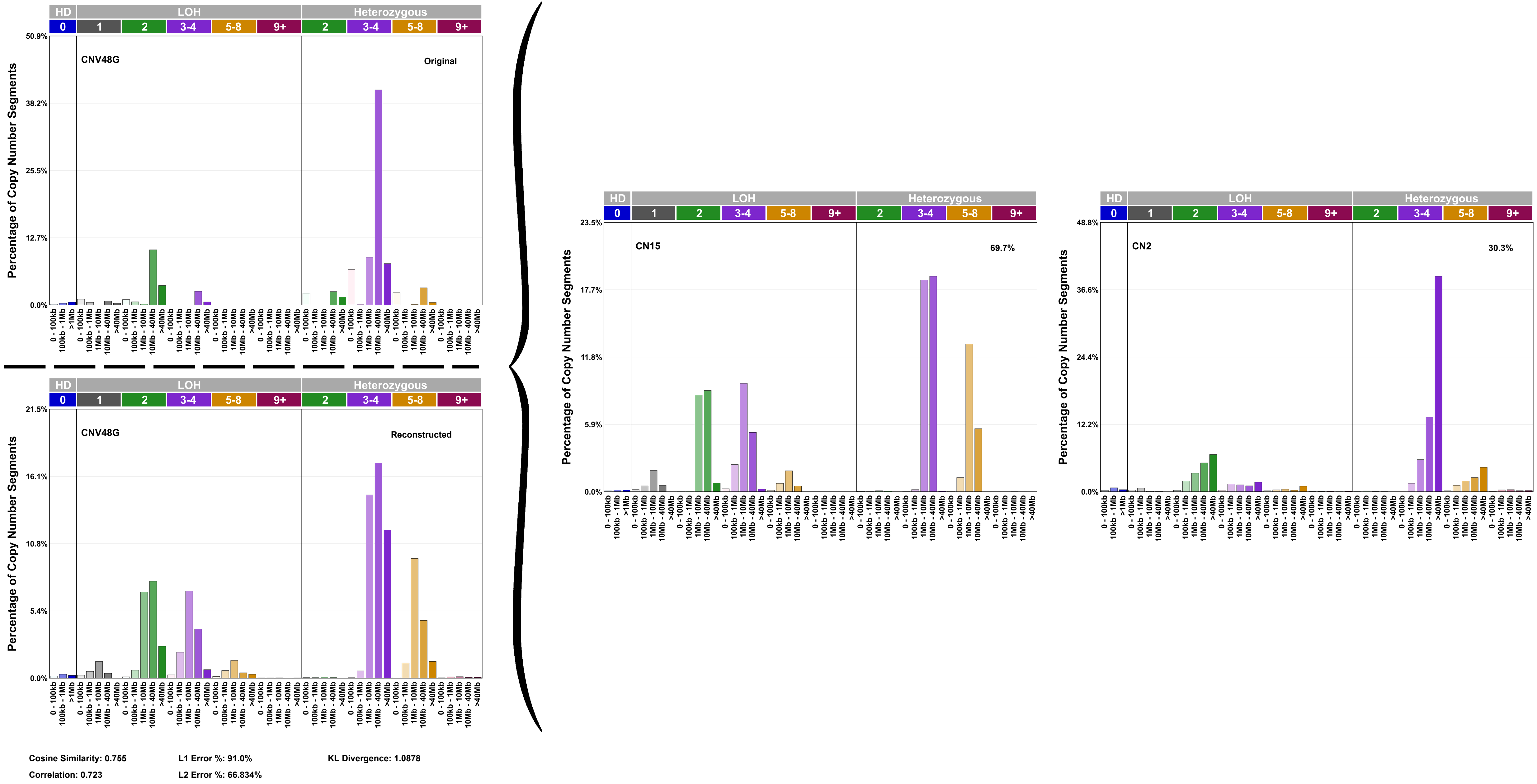
HD
LOH
Heterozygous
0
1
2
3-4
5-8
9+
Percentage of Copy Number Segments
CNV48G
Original
Reconstructed
CN15
69.7%
CN2
30.3%
0 - 100kb
100kb - 1Mb
>1Mb
1Mb - 10Mb
10Mb - 40Mb
>40Mb
50.9%
38.2%
25.5%
12.7%
0.0%
21.5%
16.1%
10.8%
5.4%
23.5%
17.7%
11.8%
5.9%
48.8%
36.6%
24.4%
12.2%
Cosine Similarity: 0.755
Correlation: 0.723
L1 Error %: 91.0%
L2 Error %: 66.834%
KL Divergence: 1.0878

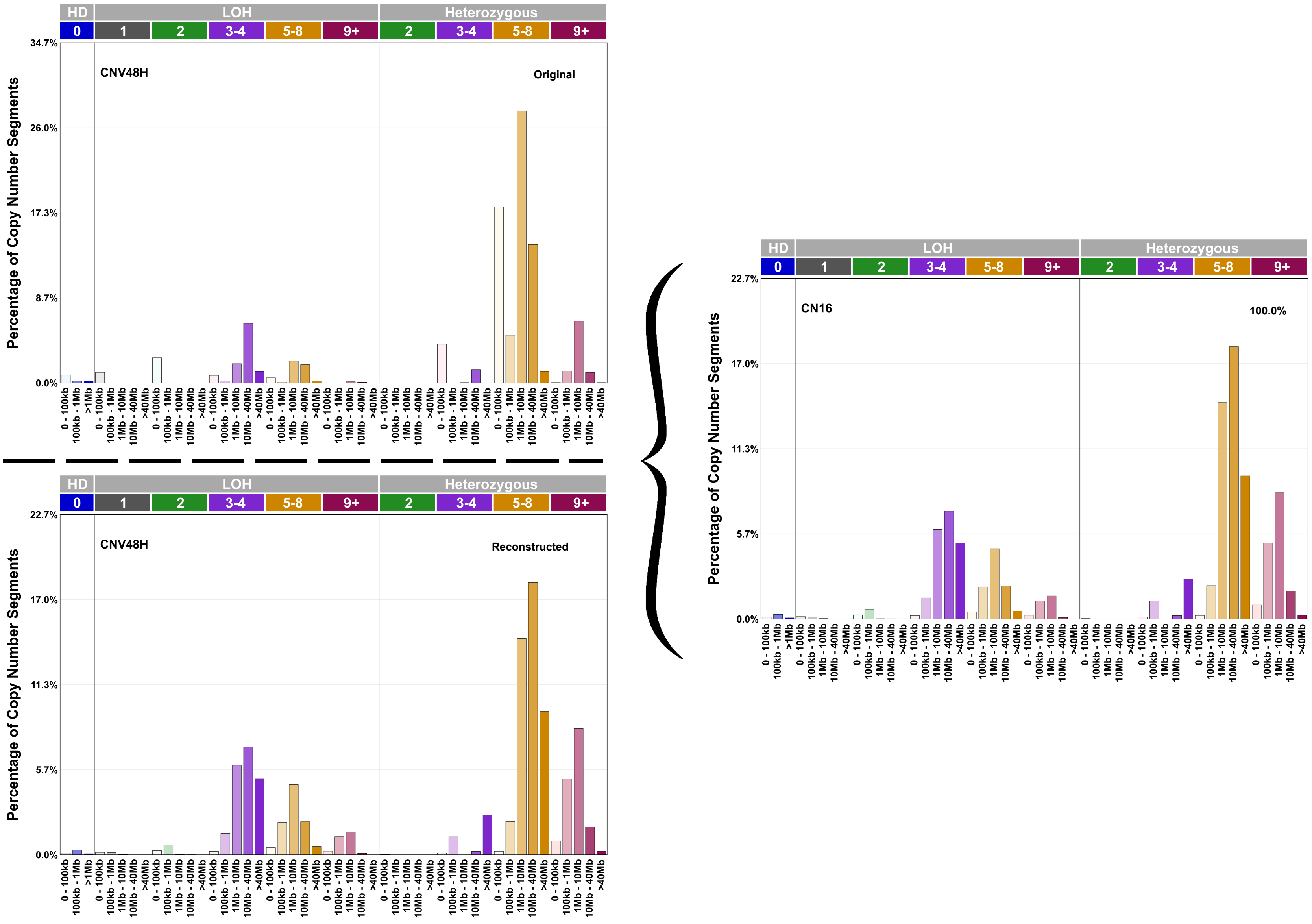


Cosine Similarity: 0.726

L1 Error %: 87.0%

KL Divergence: 1000.0

Correlation: 0.669

L2 Error %: 68.644%

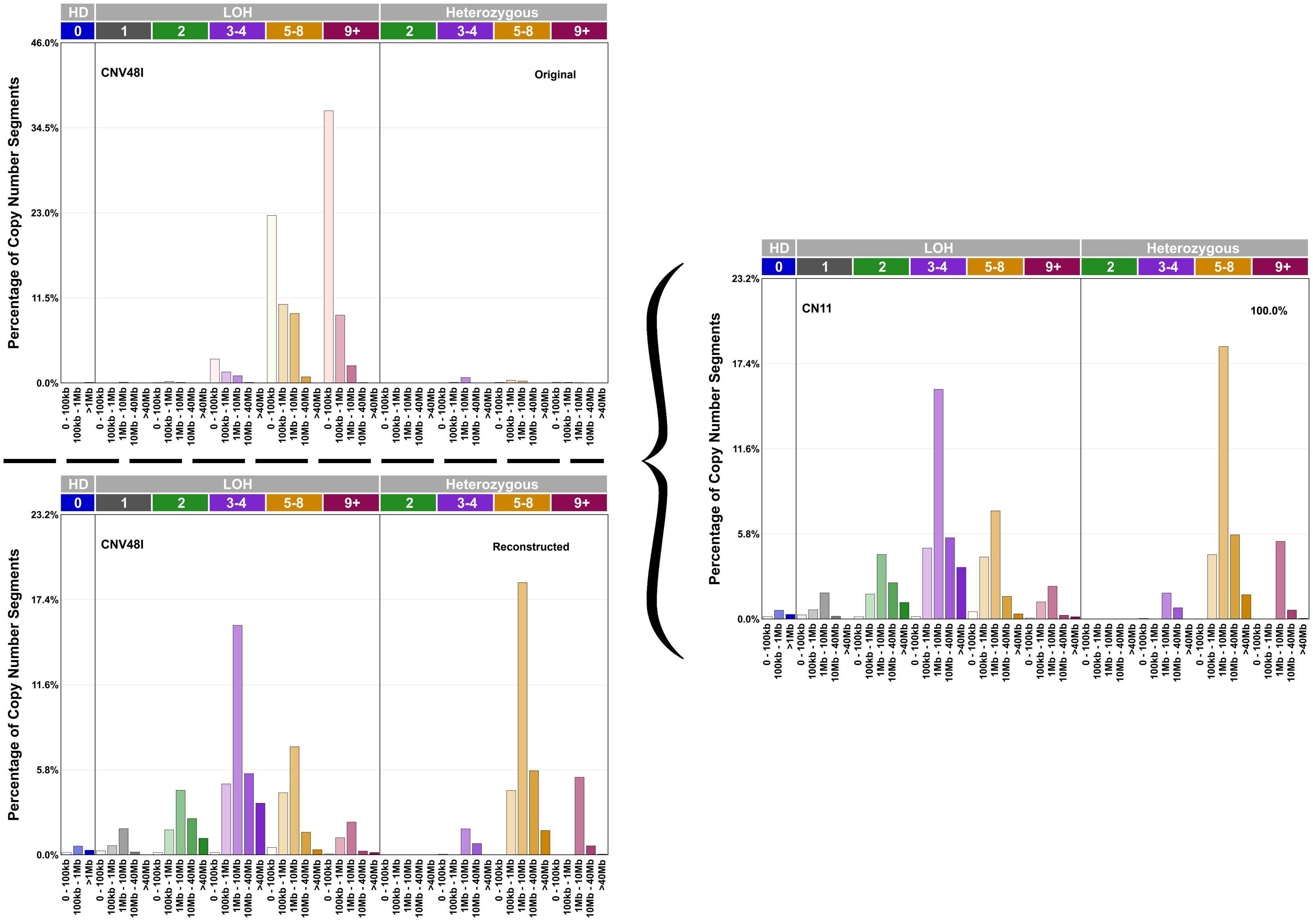


**Cosine Similarity: 0.129** **L1 Error %: 158.0%** **KL Divergence: 1000.0**

**Correlation: -0.028** **L2 Error %: 111.445%**

c

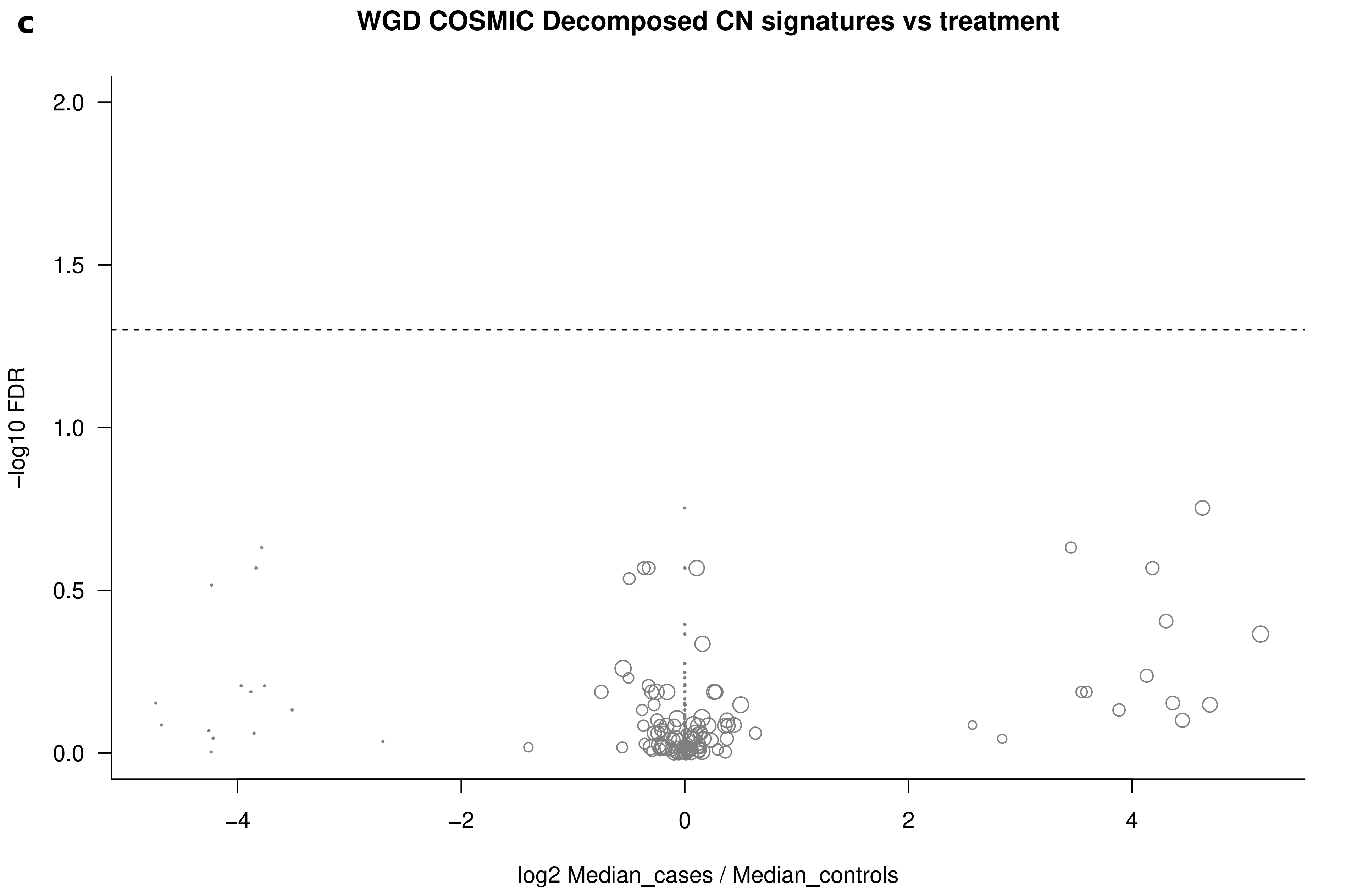

WGD COSMIC Decomposed CN signatures vs treatment
−log10 FDR
2.0
1.5
1.0
0.5
0.0
−4
−2
0
2
4
log2 Median_cases / Median_controls
