# Supplementary figures and images for: Copy number footprints of platinum-based anticancer therapies
Source: PLoS Genet. 2023 Feb 13;19(2):e1010634. doi: 10.1371/journal.pgen.1010634 (PMC9956877; doi:10.1371/journal.pgen.1010634)

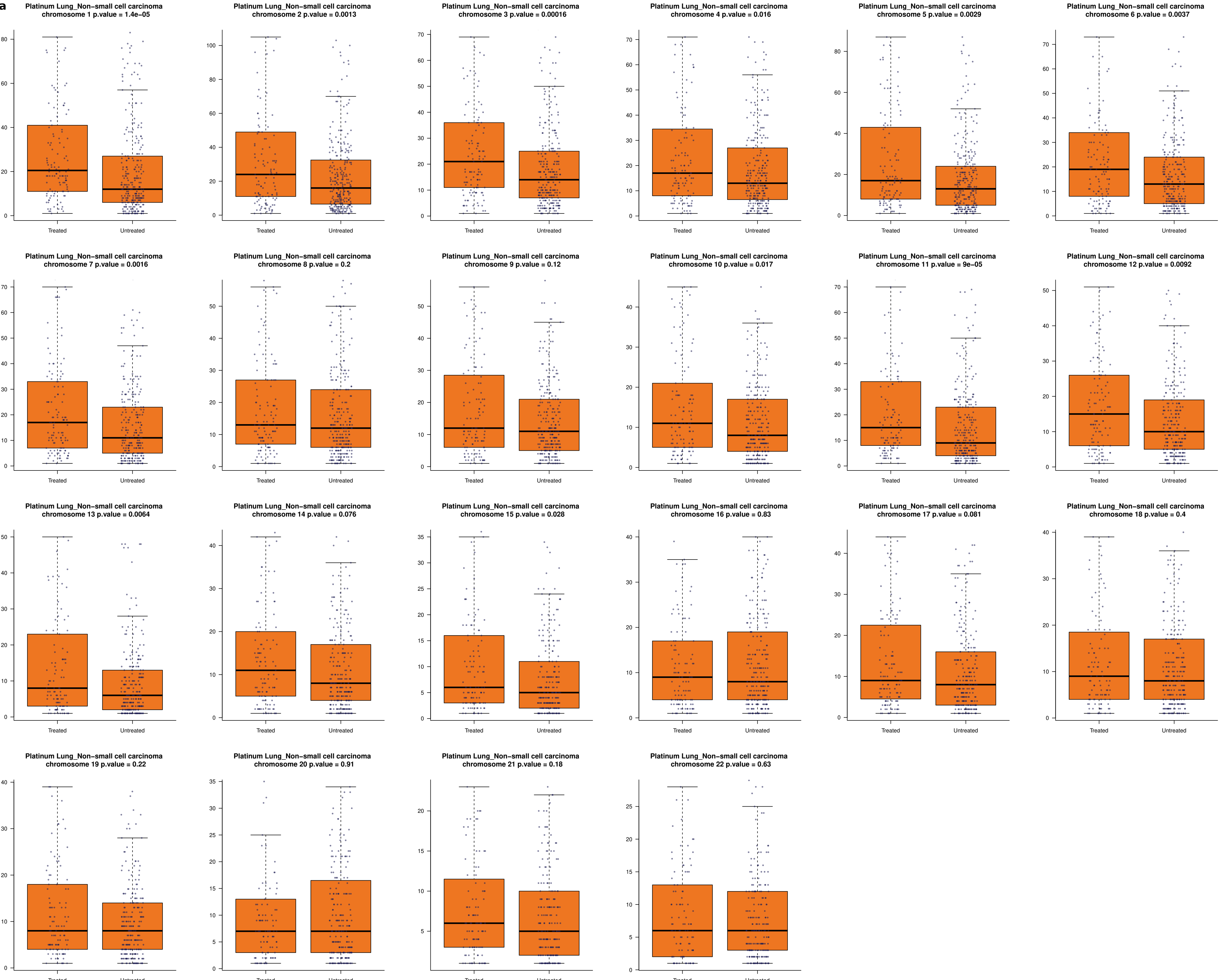

b

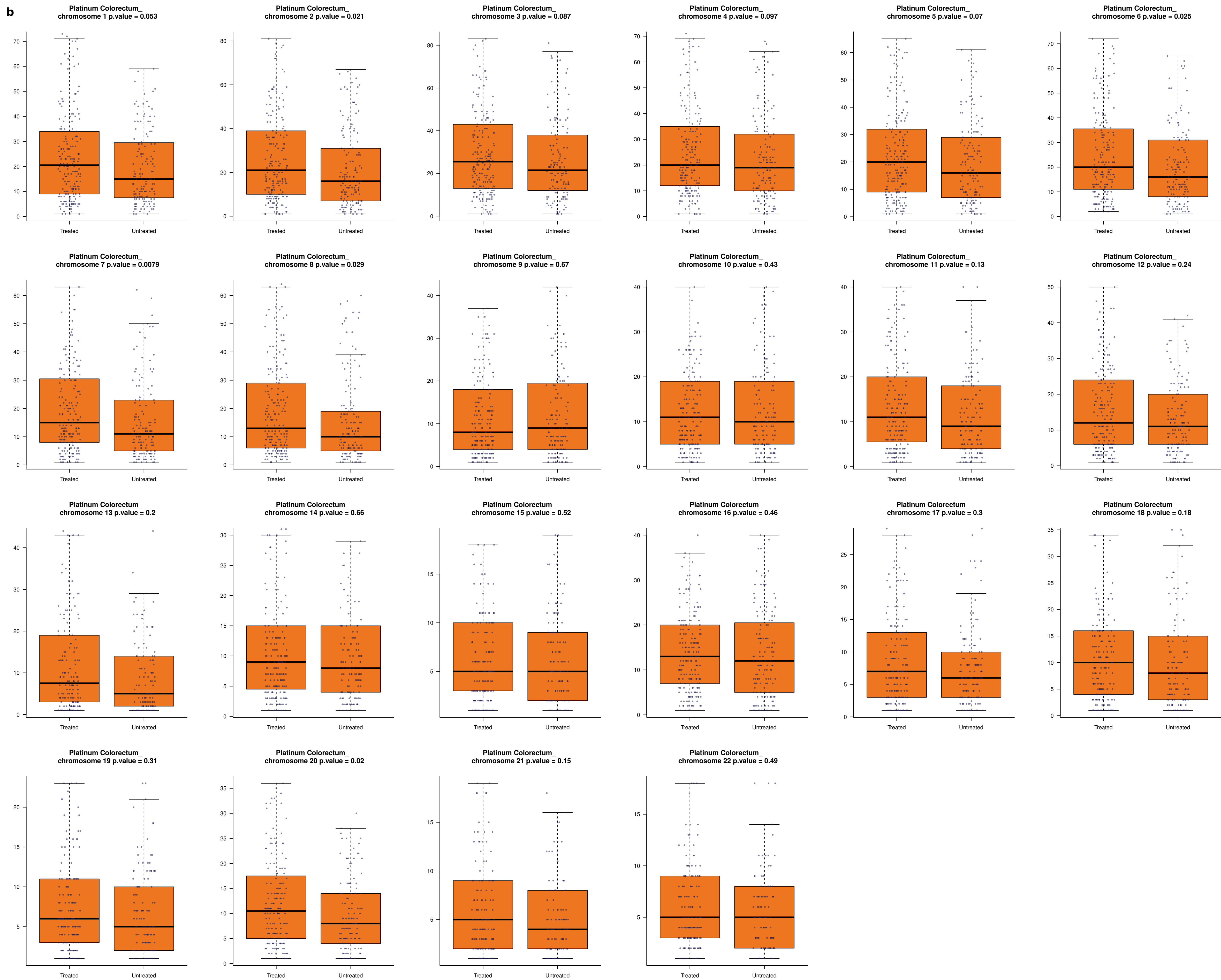

Supplement: S4 Fig — Number of chromosomal fragments with copy number 1–4 and length smaller than 10 Mb across WGD lung (a) and colorectal (b) tumors exposed or unexposed to platinum-based drugs. Individual points represent the number of chromosomal fragments in a chromosome in an exposed or unexposed tumor. P-values represent the significance of a two-tailed Wilcoxon-Mann-Whitney test. (PDF) [file pgen.1010634.s004.pdf]

**a**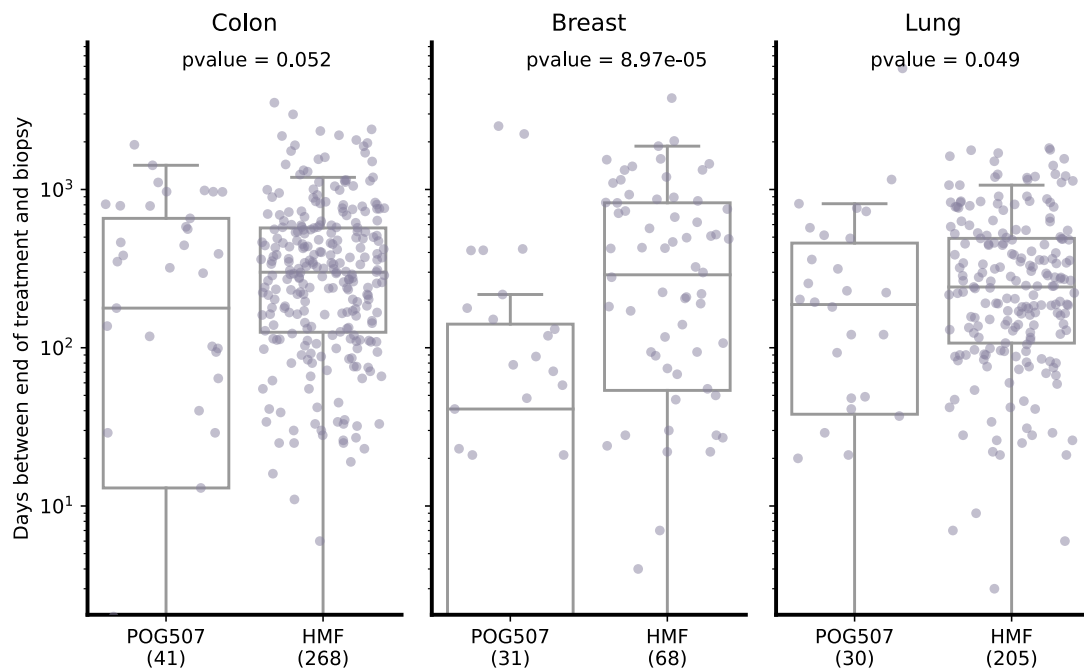**b**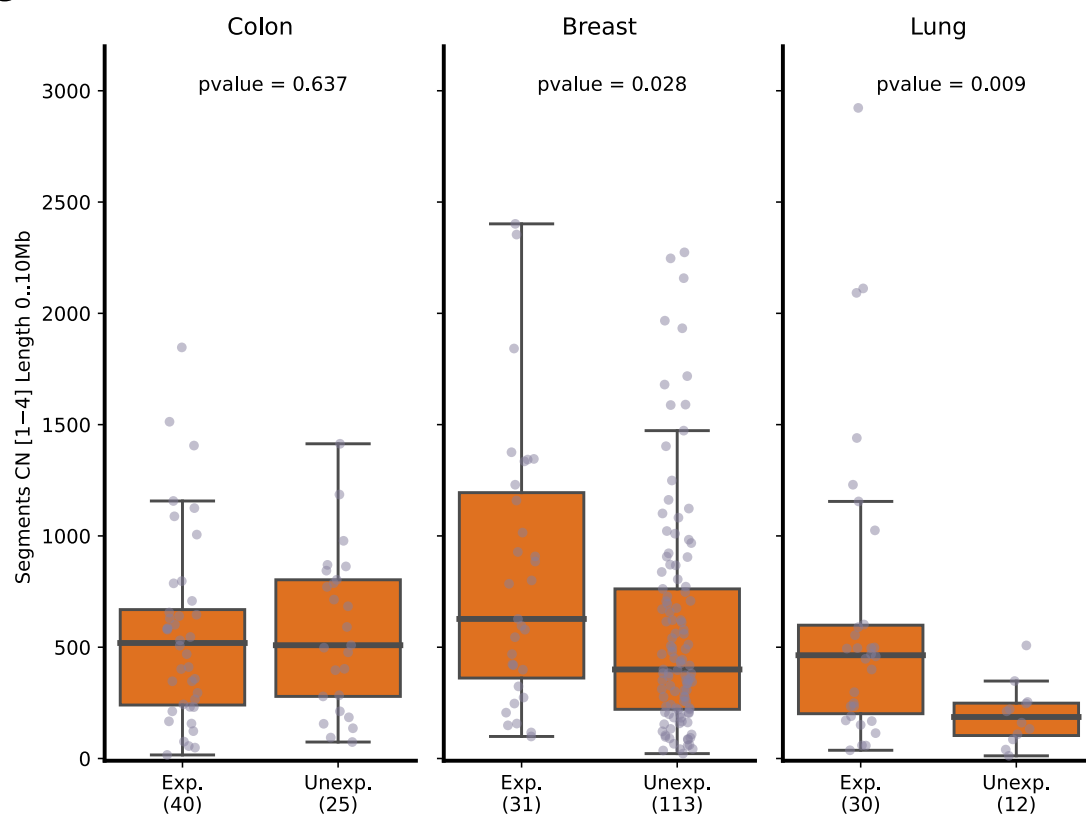

Supplement: S7 Fig — a) Days elapsed between the end of platinum treatment and the biopsy of metastatic or recurrent tumors across the POG570 and HMF cohorts. Significantly longer time lapses for breast and lung tumors (and close to significant for colorectal tumors) are apparent across the HMF cohort. As a result, there is a higher likelihood of clonal expansion between treatment and biopsy (thus increasing the probability to detect platinum-related SBS and CN) in HMF metastatic tumors. This, together with the larger sample size probably explains why the platinum CN footprint is not as clear across POG570 colon tumors. One platinum exposed colorectal patient in the POG570 cohort did not have available CN data. b) Number of chromosomal fragments of size below 10 Mb identified across platinum-exposed and unexposed colon, breast and lung tumors. These numbers are significantly greater across exposed breast and lung tumors (two-tailed Wilcoxon-Mann-Whitney) and slightly greater (although not significant) across colon tumors. The platinum CN footprint is thus replicated in the POG570 cohort, except for colon tumors. (PDF) [file pgen.1010634.s007.pdf]
