# Supplementary material for: Copy number footprints of platinum-based anticancer therapies
Source: PLoS Genet. 2023 Feb 13;19(2):e1010634. doi: 10.1371/journal.pgen.1010634 (PMC9956877; doi:10.1371/journal.pgen.1010634)
Supplement: S5 Fig — The distributions of both groups of tumors from each organ are compared using a two-tailed Wilcoxon-Mann-Whitney test. (PDF) [file pgen.1010634.s005.pdf]

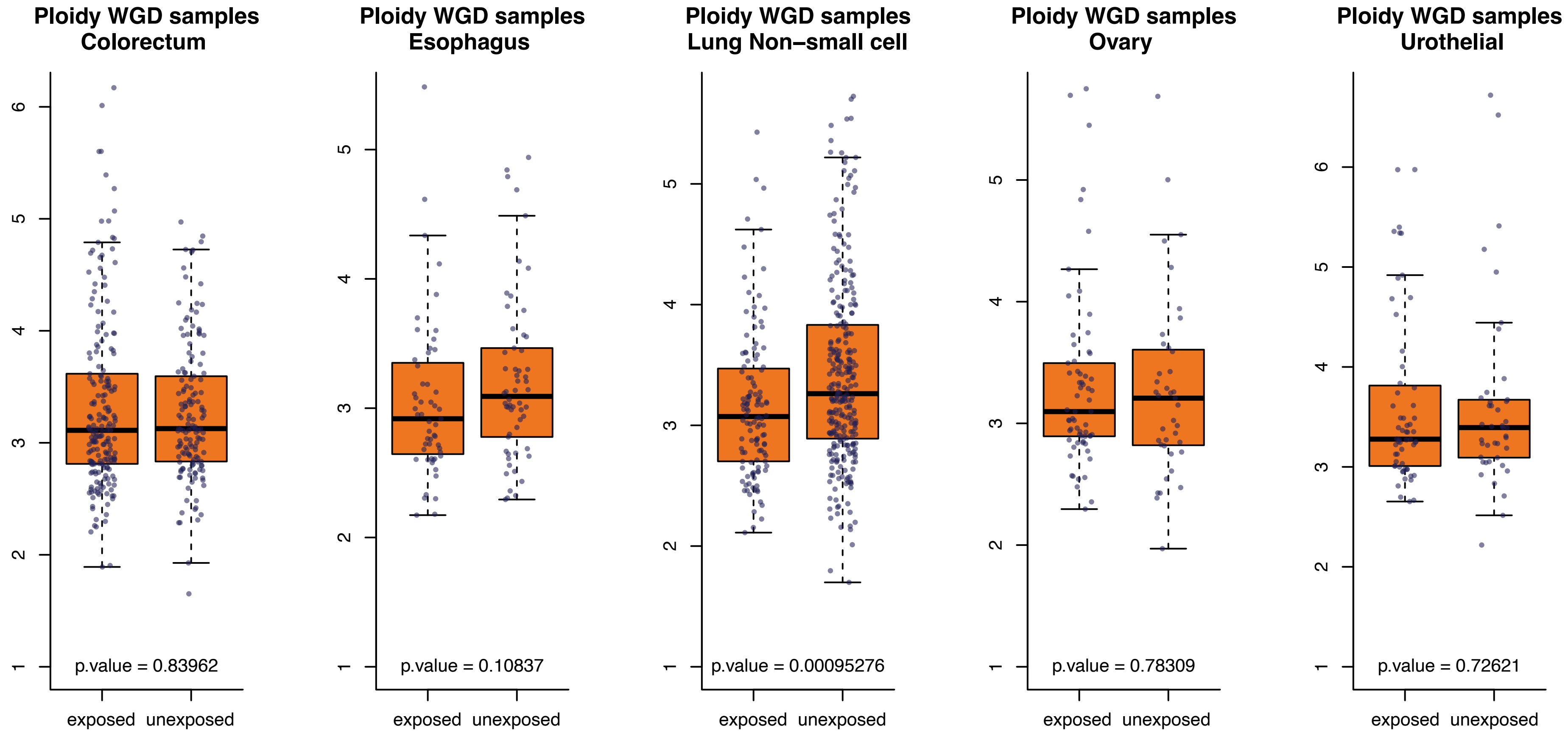

Ploidy WGD samples
Colorectum
p.value = 0.83962
Ploidy WGD samples
Esophagus
p.value = 0.10837
Ploidy WGD samples
Lung Non–small cell
p.value = 0.00095276
Ploidy WGD samples
Ovary
p.value = 0.78309
Ploidy WGD samples
Urothelial
p.value = 0.72621
exposed
unexposed
