# Supplementary material for: Copy number footprints of platinum-based anticancer therapies
Source: PLoS Genet. 2023 Feb 13;19(2):e1010634. doi: 10.1371/journal.pgen.1010634 (PMC9956877; doi:10.1371/journal.pgen.1010634)
Supplement: S8 Fig — Each curve represents a group of patients bearing either platinum-exposed or unexposed and WGD or non WGD tumors. The p-values were obtained using a Kaplan-Meier test. (PDF) [file pgen.1010634.s008.pdf]

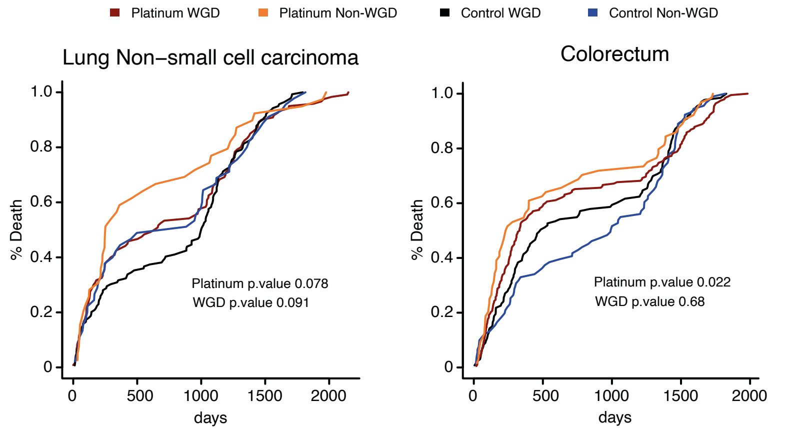

Platinum WGD
Platinum Non-WGD
Control WGD
Control Non-WGD
Lung Non−small cell carcinoma
Colorectum
% Death
days
0
0.2
0.4
0.6
0.8
1.0
0
500
1000
1500
2000
Platinum p.value 0.078
WGD p.value 0.091
Platinum p.value 0.022
WGD p.value 0.68
