# Supplementary material for: Copy number footprints of platinum-based anticancer therapies
Source: PLoS Genet. 2023 Feb 13;19(2):e1010634. doi: 10.1371/journal.pgen.1010634 (PMC9956877; doi:10.1371/journal.pgen.1010634)
Supplement: S6 Fig — (PDF) [file pgen.1010634.s006.pdf]

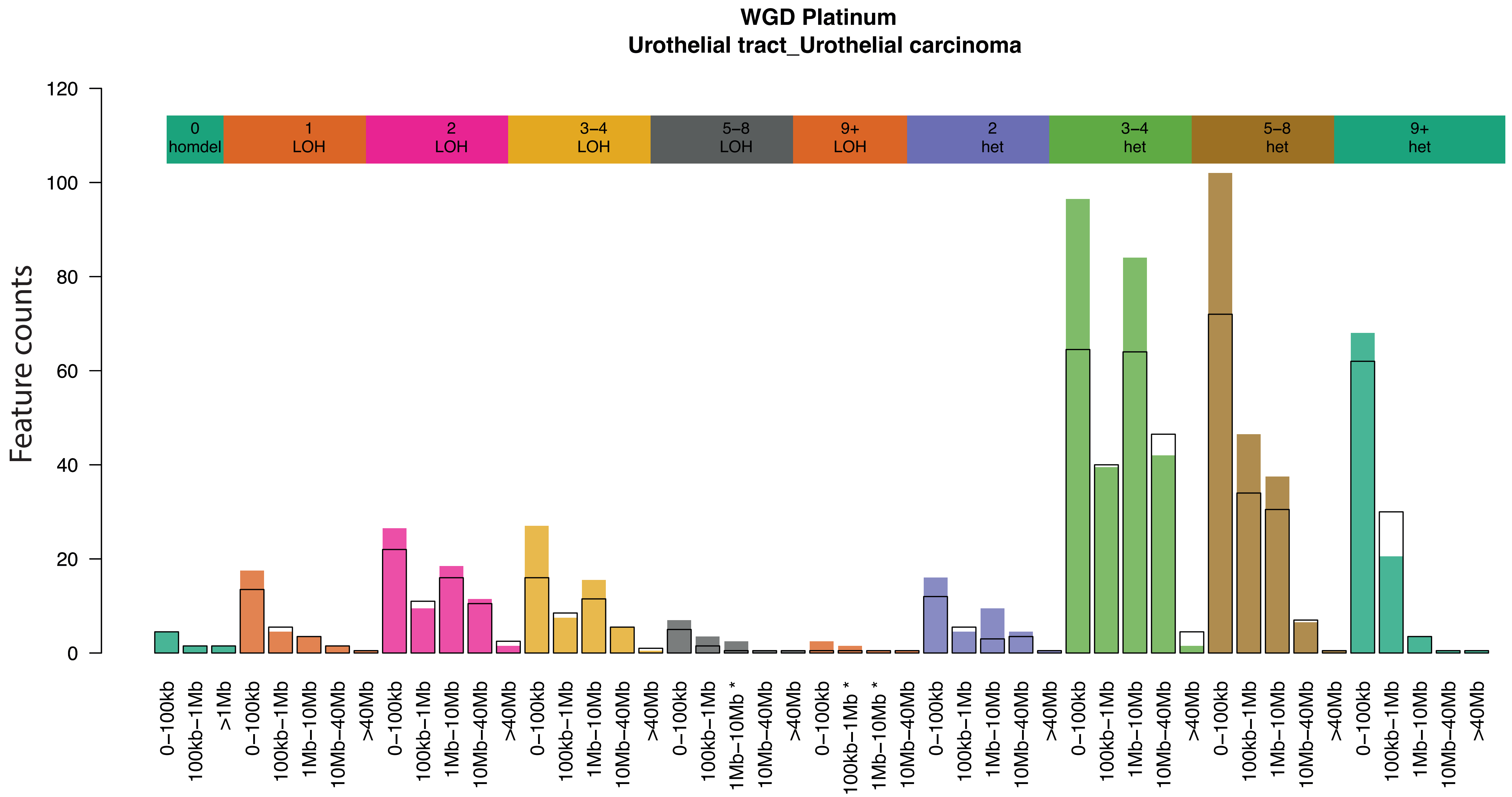
WGD Platinum
Urothelial tract_Urothelial carcinoma
Feature counts
0 homdel
1 LOH
2 LOH
3–4 LOH
5–8 LOH
9+ LOH
2 het
3–4 het
5–8 het
9+ het
0–100kb
100kb–1Mb
>1Mb
1Mb–10Mb
10Mb–40Mb
>40Mb
1Mb–10Mb *
100kb–1Mb *

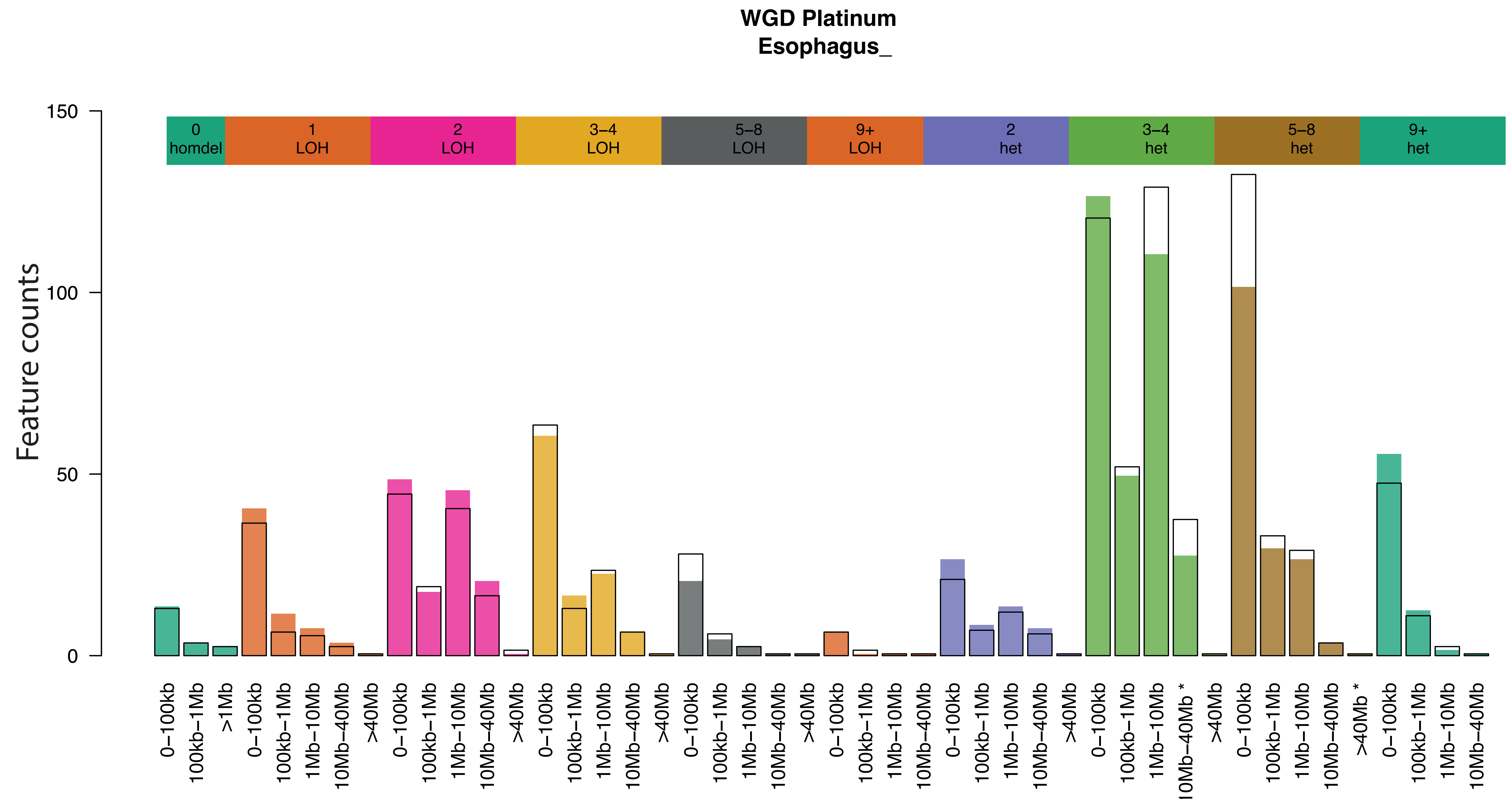
WGD Platinum
Esophagus_
Feature counts
0 homdel
1 LOH
2 LOH
3–4 LOH
5–8 LOH
9+ LOH
2 het
3–4 het
5–8 het
9+ het
0–100kb
100kb–1Mb
>1Mb
1Mb–10Mb
10Mb–40Mb
>40Mb
10Mb–40Mb *
>40Mb *
